# YY1 Suppresses Tumor Growth by Negatively Regulating the Expression and Translation Activity of eIF4E in Nasopharyngeal Carcinoma

**DOI:** 10.3390/cancers18203257

**Published:** 2026-10-09

**Authors:** Yumei Duan, Xiangting Zhou, Jianxia Wei, Changning Xue, Lemei Zheng, Ting Zeng, Hongyu Deng, Songqing Fan, Wei Xiong, Ming Zhou, Shuping Peng, Mengna Li

**Affiliations:** 1 NHC Key Laboratory of Carcinogenesis, Hunan Key Laboratory of Oncotarget Gene, Hunan Cancer Hospital and the Affiliated Cancer Hospital of Xiangya School of Medicine, Central South University, Changsha 410078, China; duanyumei@csu.edu.cn (Y.D.); zhouxiangting2026@csu.edu.cn (X.Z.); weijianxia668@163.com (J.W.); 236501044@csu.edu.cn (C.X.); 246501048@csu.edu.cn (L.Z.); 246511080@csu.edu.cn (T.Z.); denghongyu@hnca.org.cn (H.D.); xiongwei@csu.edu.cn (W.X.); zhouming2013@csu.edu.cn (M.Z.); shuping@csu.edu.cn (S.P.); 2 Cancer Research Institute and School of Basic Medical Sciences, Central South University, Changsha 410078, China; 3 The Key Laboratory of Carcinogenesis and Cancer Invasion of the Chinese Ministry of Education, Central South University, Changsha 410078, China; 4 Department of Pathology, Xiangya Hospital, Central South University, Changsha 410008, China; 5 Department of Pathology, The Second Xiangya Hospital, Central South University, Changsha 410011, China; songqingfan@csu.edu.cn

**Keywords:** nasopharyngeal carcinoma, YY1, eIF4E, 4E-BP1, eIF4G, translation activity, tumor growth

## Abstract

**Background:** Nasopharyngeal carcinoma (NPC) is a complex malignancy, and the molecular mechanisms that drive its progression have not yet been fully elucidated. Eukaryotic translation initiation factor 4E (eIF4E), a pivotal regulator of protein synthesis, is frequently dysregulated in cancers. We previously identified eIF4E as a direct transcriptional target of Yin Yang 1 (YY1) in an axis where YY1 suppressed NPC invasion and metastasis. **Methods**: Here, we investigated the function of eIF4E in NPC growth and its regulatory relationship with YY1. The CCK-8 assay, clonogenic survival assay, Annexin V/propidium iodide assay, and xenograft models were used to determine the role of YY1/eIF4E in NPC growth; Western blotting and Co-IP were performed to examine protein expression and protein–protein interactions, respectively. **Results:** Our findings demonstrate that eIF4E potently promoted NPC cell proliferation, accelerated cell cycle progression, and inhibited apoptosis. Moreover, these effects were critically dependent on its phosphorylation at serine 209 (S209). Furthermore, we uncovered a novel regulatory mechanism whereby YY1 enhances the association between 4E-BP1 and eIF4E, downregulates eIF4G expression, and is associated with reduced interaction between eIF4E and eIF4G, thereby potentially limiting eIF4F complex formation. **Conclusions:** This study establishes eIF4E phosphorylation as an oncogenic driver of NPC cell growth and survival and identifies YY1 as a tumor suppressor that negatively regulates this axis. These insights position eIF4E as a promising and precise therapeutic target for NPC intervention.

## Simple Summary

We previously reported that the transcription factor YY1 exerted tumor-suppressive functions in nasopharyngeal carcinoma (NPC), where eIF4E acted as a direct downstream transcriptional target, and YY1 repressed NPC invasion and metastasis by modulating the eIF4E/Snail/E-cadherin signaling cascade. Building on these findings, we now extend our investigation to the role of eIF4E in NPC cell proliferation and tumorigenesis. We demonstrate that eIF4E is aberrantly upregulated in NPC cells. Functional assays reveal that elevated eIF4E expression drives cell proliferation, promotes G1/S cell cycle transition, and confers cells’ resistance to apoptosis. Importantly, we identify a previously unrecognized regulatory mechanism whereby YY1 attenuates eIF4E-dependent translation initiation, accompanied by enhanced 4E-BP1-eIF4E association and reduced eIF4E-eIF4G interaction, thereby limiting eIF4F complex formation. These findings indicate that YY1 suppresses NPC growth by negatively regulating both eIF4E expression and its translational function.

## 1. Introduction

Nasopharyngeal carcinoma (NPC) is an epithelial malignant tumor that originates from the top and lateral walls of the nasopharynx. It has high incidence rates in Southeast Asia and southern China, with noticeable regional clustering and ethnic predisposition [1]. Currently, it is acknowledged that NPC is a complex disease caused by multiple factors, such as Epstein–Barr virus infection, various gene mutations, and environmental changes [2]. NPC is characterized by high malignancy, ease of metastasis, difficult early diagnosis, and complex pathogenesis [3]. Although significant progress has been made in the diagnosis, treatment, and prognosis of NPC patients in recent years [4,5,6], the complex mechanism of occurrence and development remains the main reason for the difficulty in diagnosis, low survival rate, and high mortality that affect the population. Therefore, exploring new mechanisms of progression and identifying key target molecules remain the forefront and hot spots in the current research field of NPC.

YY1 was first described as a transcription factor in 1991 [7]. Due to its dual functions of activating and inhibiting transcription, it is also known as “Yin–yang 1”. YY1 is one of the common multifunctional zinc finger transcription factor members existing in the zinc finger transcription factor histone families GLI-Kruppel and PcG [8]. Zinc finger proteins are transcription factors with finger-like domains that regulate gene expression at the transcriptional and translational levels by binding to specific sequences of target molecules, including DNA, RNA, and other zinc finger proteins. YY1 plays a significant role in various human life processes, such as cell growth, cell differentiation, cell proliferation, and immune responses, with different biological significance. In recent years, YY1 has been researched for its abnormal expression in a variety of tumors, and its significant tissue specificity in different tissues or cells has been confirmed. It can exert dual biological functions of promoting or inhibiting cancer progression in different tumors by regulating different downstream target genes or signaling pathways, activating or inhibiting gene expression [9,10,11,12]. However, its role in NPC has rarely been reported.

Eukaryotic initiation factor 4E (eIF4E), a cap-binding protein, plays a crucial role in the initiation of protein translation. eIF4E is highly expressed in approximately 30% of tumor types, including gastric cancer [13], NPC [14], head and neck squamous cell carcinoma [15], colon cancer [16], prostate cancer [17], cervical cancer [18], and lung cancer [19], and is positively correlated with the malignant progression and poor prognosis of tumors. Moreover, the overexpression of eIF4E can significantly alter the morphology of tumor cells and induce tumor occurrence, proliferation, invasion, EMT, and metastasis [20,21,22]. Therefore, eIF4E is an oncogene closely related to multiple tumors and participates in tumor occurrence and malignant development.

In many cellular and pre-clinical models of cancers, the deregulation of eIF4F leads to increased translation of specific mRNA subsets that are involved in cancer proliferation and survival. eIF4F is a hetero-trimeric complex built from the cap-binding subunit eIF4E, the helicase eIF4A, and the scaffolding subunit eIF4G. Critical to the assembly of active eIF4F complexes is the protein–protein interaction between eIF4E and eIF4G [23]. On the other hand, 4E-BPs and eIF4G compete for binding to the eIF4E dorsal surface via a shared canonical 4E-binding motif and also contain auxiliary eIF4E-binding sequences, which are assumed to come into contact with non-overlapping eIF4E surfaces [24]. Interestingly, eIF4E-dependent genes such as cyclin D1 and c-Myc are mostly linked with growth-promoting, oncogenic functions [25,26].

Our previous studies have confirmed that YY1 directly negatively regulates eIF4E, which inhibits cell migration, invasion, EMT progression, and in vivo lung metastasis in NPC [27]. Herein, with a series of experiments, we demonstrate that eIF4E, which is directly negatively regulated by YY1, promotes NPC cell proliferation and in vivo tumor growth upon the activation of eIF4E at serine 209 (S209). Moreover, restoring eIF4E expression effectively reverses the YY1-mediated inhibition of NPC cell proliferation and xenograft tumor growth. Moreover, YY1 is suggested to reduce the phosphorylation of 4E-BP1 at Thr37/46 and Ser65 without substantially affecting total 4E-BP1 expression, thereby reducing the downstream eIF4E translation process in the cytoplasm. At the same time, it can also downregulate the expression of eIF4G, which inhibits the formation of the translation initiation complex eIF4F. The combined effect mentioned above significantly inhibits the oncogenic role of eIF4E in NPC cells. Collectively, our findings provide a further scientific basis and potential therapeutic targets for NPC diagnosis and treatment.

## 2. Materials and Methods

### 2.1. Cell Culture

The NPC cell lines 5-8F and HNE2 used in this study were maintained in our laboratory at the Cancer Research Institute of Central South University. The cells were cultured in DMEM complete medium supplemented with 10% fetal bovine serum and 1% penicillin/streptomycin dual antibiotics (Biological Industries, Jerusalem, Israel). The culture environment of the cells was a 37 °C constant-temperature incubator containing 5% CO_2_. Furthermore, we constructed NPC cell models overexpressing YY1 and eIF4E by using both the 5-8F and HNE2 cell lines.

### 2.2. Plasmid Construction and Stable Cell Lines

YY1-overexpressing 5-8F and HNE2 cells were established as previously described [17] using a pCDH-copGFP lentiviral vector encoding full-length human YY1 with a 3 × FLAG tag. For eIF4E overexpression, pCMV-HA-eIF4E-WT was constructed by inserting the full-length human eIF4E coding sequence into the pCMV-HA vector. The pCMV-HA-eIF4E-S209A mutant was generated by site-directed mutagenesis, replacing S209 with alanine, and was verified by Sanger sequencing.

### 2.3. RNA Interference and Transfection

As mentioned in the previous experiments, the specific small interfering RNA (siRNA) for eIF4E was purchased from Ribobio (Ribobio, Guangzhou, China). The siRNA sequences were as follows (5′-3′): siRNA eIF4E #1, TGGCGCTGTTGTTAATGTT; siRNA eIF4E #2, AGAGCTAAAGGTGATAAGA. To knock down the expression of eIF4E in cells, we used lipo3000 (Invitrogen/Thermo Fisher Scientific, Carlsbad, CA, USA) to construct a cell model by transfecting exponentially growing cells with siRNA, according to the manufacturer’s instructions.

### 2.4. Protein Extraction and Western Blot

Cell proteins were extracted after the cells were collected and digested using cell lysate (NCM Biotech, Suzhou, China), and the BCA Protein Assay kit was used (Thermo Fisher Scientific, Waltham, MA, USA) to determine the concentration before the gel electrophoresis experiment was carried out. The loading amount of each sample was approximately 20–30 µg. Denatured proteins were separated on 8–12% SDS gels and then transferred onto a 0.45 µm PVDF membrane (Millipore, Billerica, MA, USA). Then, the membrane was blocked with 5% skimmed milk at room temperature for about 60 min and incubated overnight with the corresponding primary antibody at 4 °C. The following primary antibodies were used: anti-YY1 (Proteintech, Wuhan, China; #66281-1-Ig; 1:2000), anti-eIF4E (Proteintech, Wuhan, China; #66655-1-Ig; 1:2000), anti-eIF4E (phospho S209) (Abcam, Cambridge, UK; ab76256; 1:2000), anti-4E-BP1 (Abclonal, Wuhan, China; #A24691; 1:1000), anti-eIF4G (Abclonal, Wuhan, China; #A7552; 1:1000), Phospho-4E-BP1-Thr37/46 (Abclonal, Wuhan, China; #AP0030; 1:1000), anti-p-4E-BP1-Ser65 (Selleck, Houston, TX, USA; #F418201; 1:1000), anti-p-p70 S6 Kinase (Thr389) (Cell Signaling Technology (CST), Boston, MA, USA; #9234; 1:2000), anti-GAPDH (Proteintech, Wuhan, China; #60004-1-Ig; 1:10,000), anti-CDK4 (Abclonal, Wuhan, China; #A0366; 1:1000), anti-P21 (CST, Boston, MA, USA; #2947; 1:1000), and anti-c-PARP (CST, Boston, MA, USA; #5625; 1:1000). Then, the membrane was incubated with the corresponding secondary antibodies (Abclonal, Wuhan, China; #AS003 and AS014; 1:10,000) at 37 °C for 1 h. Protein signals were detected using the ECL luminescence system (Accurate Biotechnology, Hunan, China) and the modified chemiluminescence detection system (MiniChemi™ I, SageCreation, Beijing, China).

### 2.5. Puromycin Incorporation (SUnSET) Assay

Protein synthesis was assessed using the surface sensing of translation (SUnSET) assay based on puromycin incorporation into nascent polypeptide chains. Control, YY1-overexpressing, YY1+eIF4E-WT, and YY1+eIF4E-S209A 5-8F and HNE2 cells were incubated with puromycin (Selleck, Shanghai, China; 10 μg/mL) for 10 min. For inhibition controls, cells were treated with cycloheximide (CHX, Selleck, Shanghai, China; 100 μg/mL) to suppress protein synthesis before puromycin labeling. After treatment, cells were harvested and lysed, and equal amounts of total protein were subjected to Western blot analysis. Puromycin incorporation into newly synthesized polypeptides was detected using an anti-puromycin monoclonal antibody. YY1 and GAPDH expression levels were examined in parallel to confirm YY1 overexpression and protein loading, respectively.

### 2.6. CCK-8 Assay

We inoculated 1000 cells per well in a 96-well plate. Each experimental group was set with 5 replicates. Under light-protected conditions, 10 µL of CCK-8 reagent was added to each well. After continuous culture for 2 h, the absorbance value of the cells (at 450 nm) was determined using a microplate reader, and this time point was set to 0 h. Subsequently, 10 µL of CCK-8 reagent (Abbkine, Wuhan, China) was added at 24 h, 48 h, 72 h, and 96 h. After continued incubation for 2 h, the OD value was measured. The growth curve of the cells, where the horizontal coordinate is the time point and the vertical coordinate is the relative OD value of the corresponding group, was constructed using GraphPadPrism v9.0.

### 2.7. Colony Formation

Twenty-four hours after transfection, the cells were inoculated into six-well plates at a density of approximately 1000 cells per well, with 3 replicates set in each group. We stopped the culture when clear cell clones were visible to the naked eye after continuous culture for 14 days. After being stained with crystal violet staining solution, the clones were scanned, and the number of formed clones was measured. The number of clone formations obtained was statistically analyzed using inter-group difference analysis, and a bar chart was drawn.

### 2.8. Flow Cytometry Analysis of Cell Cycle and Cell Apoptosis

The cell cycle assay was performed using propidium iodide (PI) staining. Cells were harvested, washed, fixed, and incubated with respective reagents before flow cytometric detection. Data were analyzed using ModFit LT 5.0 to quantify the percentages of cells in G0/G1, S, and G2/M phases. All experiments were repeated at least three times independently.

The apoptosis rate in each sample was assessed using a Cell Apoptosis Kit (BD Biosciences, San Jose, CA, USA). Specifically, cells were seeded in a culture plate for 12 h and then transfected. After 48 h, the cells were collected, washed three times using PBS, and resuspended in 500 μL of binding buffer containing 5 μL of propidium iodide (PI) and 5 μL of Annexin V-FITC. Data were analyzed using FlowJo V10. We categorized the cells as follows: Viable cells = FITC^_^PI^_^; early apoptotic cells = FITC+PI^_^; and late apoptotic/necrotic cells = FITC+PI+. The apoptosis rates presented include both early and late apoptotic cells.

### 2.9. Co-Immunoprecipitation Assay

Pierce^TM^ Protein A/G Magnetic Beads (Thermo Fisher Scientific, Waltham, MA, USA) were incubated with primary antibodies for two hours at RT to promote capture, and then lysis buffer was used to break the cells; subsequently, the cells were incubated with bead–immune complexes at 4 °C overnight, rinsed using immunoprecipitation wash buffer, and eluted with loading buffer. The eluates were subsequently resolved using SDS-PAGE.

### 2.10. Subcutaneous Xenograft Model

Twenty female nude BALB/c mice aged 5 weeks were purchased for experiments in vivo. NPC cells of different groups were counted and resuspended in normal saline. The number of cells inoculated into each mouse was about 8 × 10^6^, and the volume was about 100 μL. The general condition of the mice was observed every day, and their weight and the volume of the tumor were recorded every other day. Thirty days after the injection, the mice were euthanized. The subcutaneously transplanted tumor tissues of the nude mice were separated. The general morphology and appearance of the tumor nodules were photographed, and the tumor tissues were immediately fixed and preserved with neutral formalin solution.

### 2.11. IHC Staining

Immunohistochemical staining was performed using an IHC kit (MXB Biotechnologies, Fuzhou, China) and DAB kit (20×; MXB Biotechnologies, Fuzhou, China) according to the manufacturer’s instructions. Brifly, paraffin sections with a thickness of 3 μm were dewaxed and then subjected to high-pressure repair with antigen repair solution (EDTA, pH 9.0) for 2.5 min. After inactivation and blocking, they were incubated overnight with the corresponding primary antibody. The next day, they were incubated with the corresponding secondary antibody for 30 min. After DAB staining, they were scored based on the staining intensity and area under the microscope.

### 2.12. Statistical Analysis

All the statistical analyses in this study were conducted using GraphPad v9.0. All quantitative data were expressed as mean ± standard deviation (SD). Statistical significance was evaluated with Student’s *t*-test. A *p*-value < 0.05 was considered statistically significant. All experiments were repeated at least three times.

## 3. Results

### 3.1. Overexpression of eIF4E Promoted NPC Cell Proliferation

**Figure 1:**
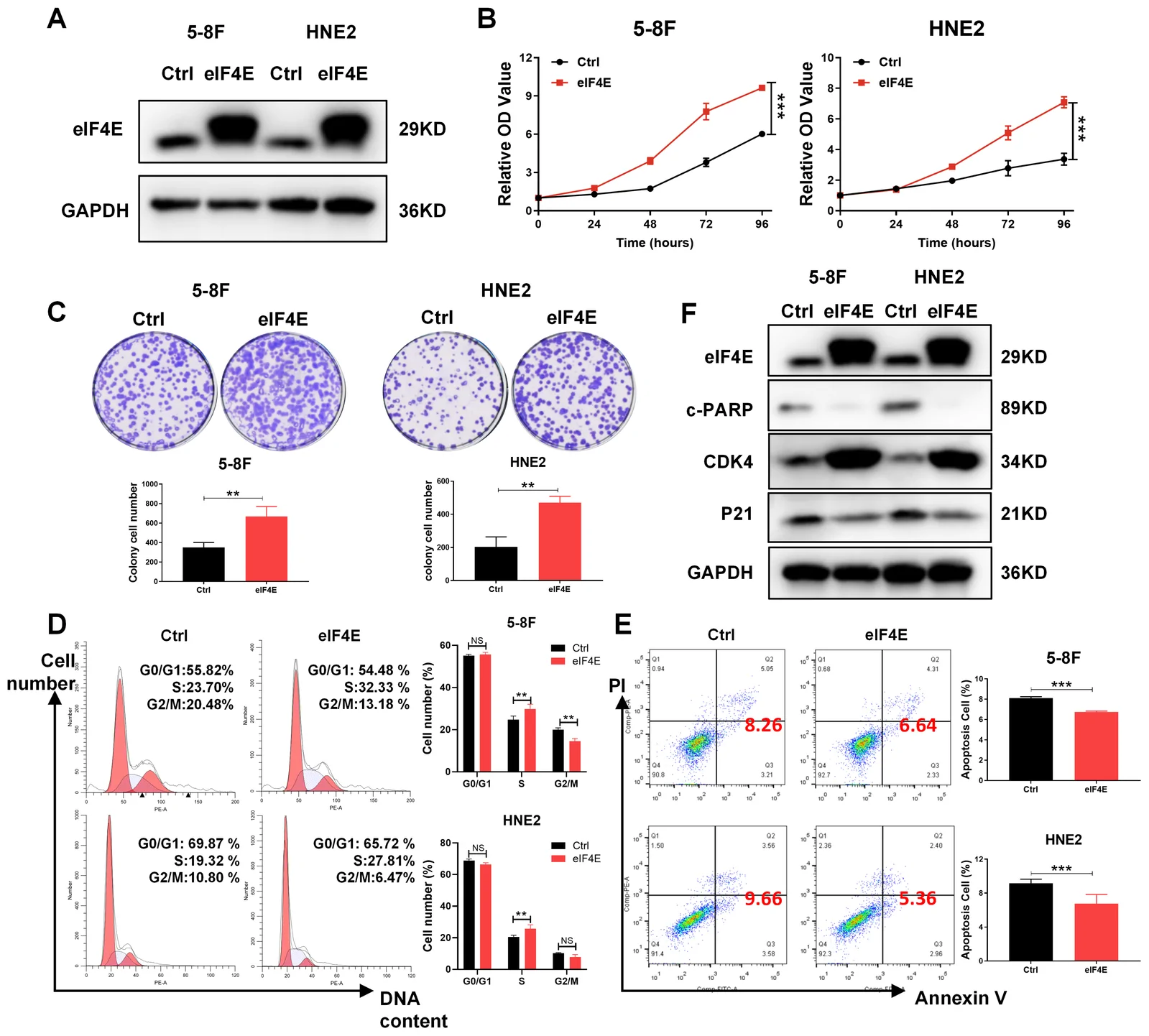
eIF4E promotes cell proliferation and functions as a candidate oncogene in NPC cells. (**A**) Western blotting was performed to detect the eIF4E protein level using an antibody against eIF4E, and GAPDH served as an internal control. (**B**) CCK-8 assay was performed to detect the cell viability of 5-8F and HNE2 cells with stable overexpression of eIF4E. (**C**) A colony-forming assay was used to detect the cell growth of 5-8F and HNE2 cells with stable overexpression of eIF4E. (**D**) Flow cytometry analysis of cell cycle distribution and the representative (left) and statistical results (right) were shown. (**E**) Flow cytometry analysis of cell apoptosis via Annexin V-FITC and PI double staining, and the representative (left) and statistical results (right) were shown. (**F**) Significantly differently expressed proteins involved in cell cycle progression (P21 and CDK4) and apoptosis (c-PARP) in eIF4E overexpression cells. Error bars denoted the mean ± SD. ** *p* < 0.01, *** *p* < 0.001; NS, not significant. All experiments were performed in triplicate. Original images of the blots are provided in File S1.

A variety of studies have explored the cancer-promoting effect of eIF4E, and it is noteworthy that in the databases of TCGA and CPTAC, its expression level in head and neck squamous cell carcinoma is significantly higher than that in normal tissues (Figure S1A,B). Moreover, the survival curve shows that the expression of eIF4E is significantly related to the prognosis of patients (Figure S1C) and shows a close correlation with different stages and grades of the tumor (Figure S1D,E). We further investigated the effect of eIF4E on the proliferation of NPC cells by constructing eIF4E-overexpressing NPC cell lines 5-8F and HNE2 (Figure 1A). The Cell Counting Kit-8 (CCK-8) assay and the colony formation assay were performed to evaluate the effect of eIF4E on the biological behavior of NPC cells. The results demonstrate that eIF4E overexpression significantly enhanced the proliferation capacity and colony-forming ability of NPC cells (Figure 1B,C). Subsequently, to further explore the regulatory role of eIF4E in NPC cell cycle progression, flow cytometry analysis was conducted. The results indicate that in the 5-8F and HNE2 cell lines overexpressing eIF4E, the proportion of cells in the S phase was notably increased (Figure 1D). These findings collectively confirm that eIF4E can significantly promote the transition of 5-8F and HNE2 cells from the G1 to the S phase. In addition, the impact of eIF4E overexpression on the apoptosis of NPC cells was examined. Propidium iodide (PI) and Annexin V double-staining results show that the number of apoptotic NPC cells in the eIF4E overexpression group was significantly lower than that in the control group (Figure 1E). These results indicate that eIF4E overexpression exerts an inhibitory effect on the apoptosis of NPC cells.

Concurrently, Western blot analysis revealed that in NPC cells overexpressing eIF4E, the expression level of cyclin-dependent kinase 4 (CDK4), a key marker of cell cycle progression, was upregulated, while the expression of P21 was downregulated. Meanwhile, the expression of cleaved poly(ADP-ribose) polymerase (c-PARP), a critical marker of apoptosis, was downregulated in NPC cells overexpressing eIF4E (Figure 1F). These results show that eIF4E enhances the growth of NPC cells.

### 3.2. Knockdown of eIF4E Promoted Apoptosis but Inhibited the Cell Cycle of NPC Cells

**Figure 2:**
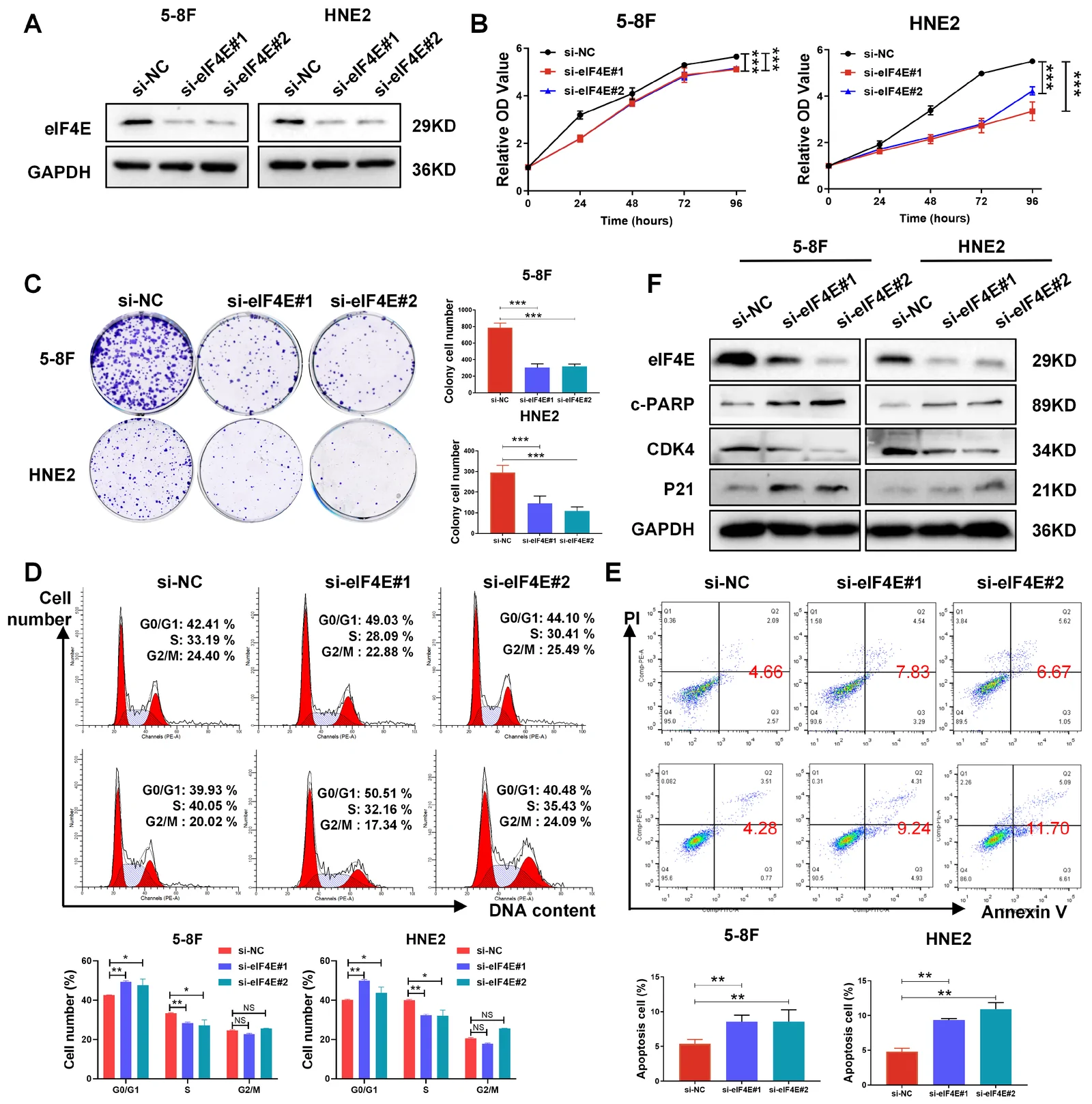
Depletion of eIF4E by siRNA inhibits cell proliferation in NPC cells. (**A**) 5-8F and HNE2 cells were transfected with scrambled siRNA (negative control) and eIF4E siRNA #1 and #2. Western blotting was used to analyze the silencing efficiency of eIF4E, and GAPDH served as an internal control. (**B**,**C**) Cell growth was measured by CCK-8 assays and colony-forming assays in 5-8F and HNE2 cells transfected with eIF4E siRNAs or a negative control. (**D**) Flow cytometry analysis of cell cycle distribution, and the representative and statistical results were shown. (**E**) Flow cytometry analysis of cell apoptosis via Annexin V-FITC and PI double staining, and the representative (up) and statistical results (down) were shown. (**F**) Significantly differently expressed proteins involved in cell cycle progression (P21 and CDK4) and apoptosis (c-PARP) in eIF4E knockdown cells. GAPDH served as an internal control. Error bars represent the mean ± SD. * *p* < 0.05, ** *p* < 0.01, *** *p* < 0.001; NS, no significant. All experiments were performed in triplicate. Original images of the blots are provided in File S1.

Furthermore, small interfering RNAs (siRNAs) targeting eIF4E were separately transfected into the NPC cell lines 5-8F and HNE2 to knockdown eIF4E expression; the experimental results are presented in Figure 2A. Subsequently, the CCK-8 assay and the cell colony formation assay were performed to validate the regulatory effect of eIF4E on NPC cell proliferation. The results confirm that eIF4E knockdown could significantly inhibit the proliferative activity of NPC cells (Figure 2B,C). The flow cytometry assay further revealed that in comparison with the control group, eIF4E silencing not only significantly blocked the progression of the cell cycle from the G1 phase to the S phase but also promoted cell apoptosis (Figure 2D,E). Concurrently, the expression levels of proliferation-related markers, including CDK4, c-PARP, and P21, exhibited significant changes (Figure 2F). Collectively, these findings consistently demonstrate that eIF4E promotes NPC cell proliferation.

### 3.3. Restoring eIF4E Levels Reverses the Effect of YY1 on Cell Proliferation and Apoptosis in NPC Cells

**Figure 3:**
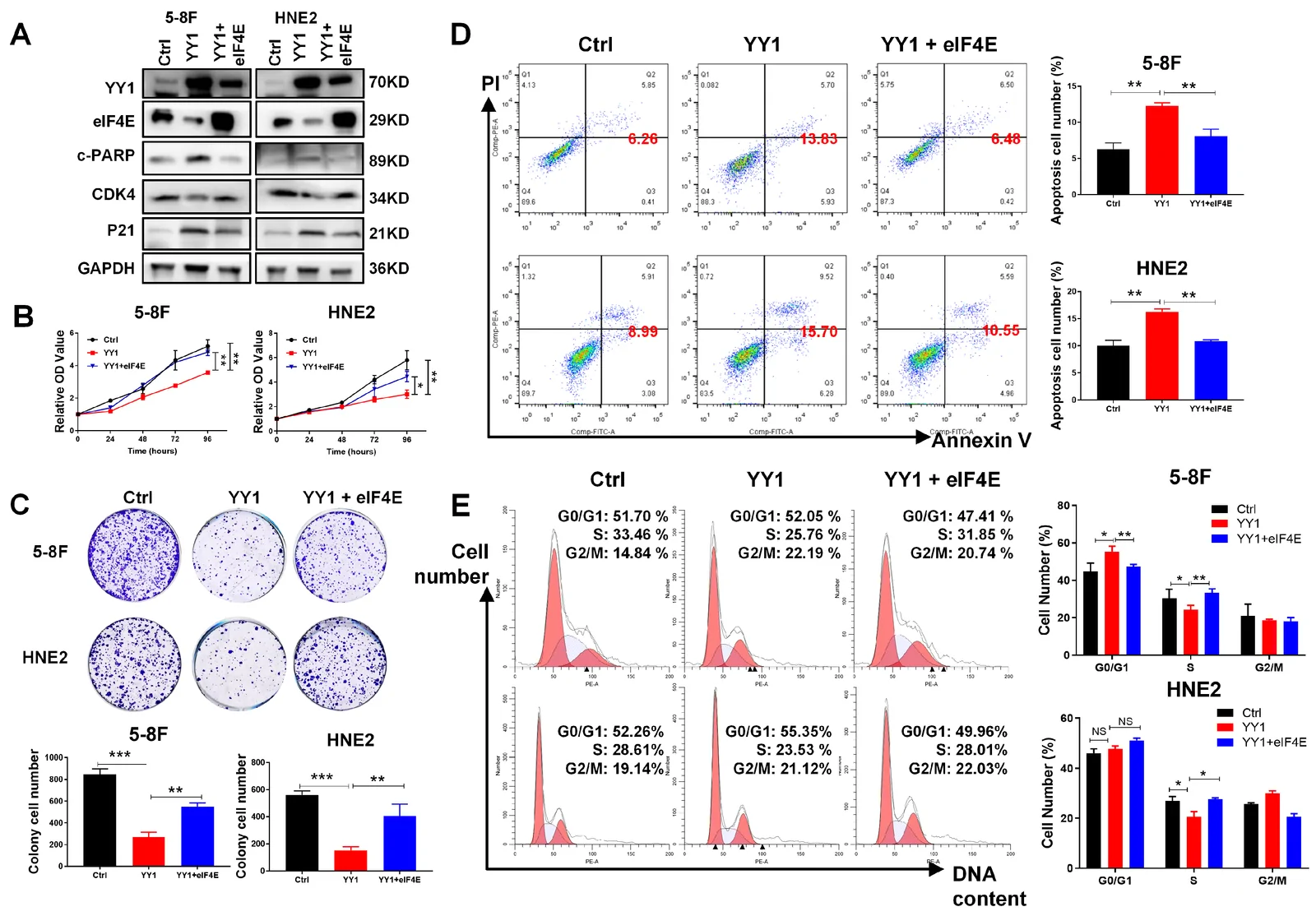
Restoring eIF4E levels reverses the effect of YY1 on cell proliferation in NPC cells. (**A**) Western blotting was used to detect the expression of YY1, eIF4E, c-PARP, CDK4, P21. GAPDH served as an internal control. (**B**) CCK-8 assays of 5-8F and HNE2 cells stably overexpressing YY1 or with restored eIF4E expression. (**C**) Colony-forming assays and quantification of colonies (1000/well). (**D**) Flow cytometry analysis of cell apoptosis via Annexin V-FITC and PI double staining. (**E**) Cell-cycle analysis was performed by flow cytometry, and the representative and statistical results were shown. Error bars represented the mean ± SD. * *p* < 0.05, ** *p* < 0.01, *** *p* < 0.001; NS, no significant. All experiments were performed in triplicate. Original images of the blots are provided in File S1.

Our previous experiments confirmed that YY1 effectively inhibited the migration, invasion, and epithelial–mesenchymal transition (EMT) of NPC cells, as well as in vivo tumor metastasis. This inhibitory effect was attributed to YY1 binding to the promoter region of eIF4E, which negatively regulates the transcriptional activity and expression of eIF4E. To further elucidate the critical role and underlying mechanism of eIF4E in YY1-mediated NPC progression, the CCK-8 and colony-forming assays were performed. As expected, the ectopic expression of YY1 significantly inhibited cell proliferation and colony formation in 5-8F and HNE2 cells. In contrast, restoring eIF4E expression in YY1-overexpressing cells markedly rescued these inhibitory effects on proliferation and colony formation compared with YY1-overexpressing cells alone (Figure 3A–C). Furthermore, YY1 overexpression significantly increased the percentage of apoptotic NPC cells compared with the negative controls, whereas restoring eIF4E expression noticeably inhibited the pro-apoptotic effect of YY1 on NPC cells (Figure 3D). Additionally, the effect of eIF4E rescue on cell cycle progression was analyzed. The results show that YY1 overexpression arrested NPC cells in the G0/G1 phase; however, restoring eIF4E expression led to a significant reduction in the G0/G1 cell population, accompanied by an increase in the S-phase cell population, compared with YY1-overexpressing cells alone (Figure 3E). Consistent with the flow cytometry results, the Western blot analysis demonstrated that YY1 overexpression significantly upregulated c-PARP and P21 expression, and downregulated CDK4 expression. Notably, restoring eIF4E expression substantially reversed these YY1-induced changes in protein expression levels (Figure 3A). Collectively, the results presented herein demonstrate that restoring eIF4E expression could at least partially abrogate the tumor-suppressive effects exerted by YY1 on the proliferation and apoptosis of NPC cells in vitro.

Previous studies have established that the oncogenic role of eIF4E is dependent on phosphorylation at serine 209 (S209). Our Western blot analysis demonstrated that YY1 overexpression reduced eIF4E phosphorylation at S209 in NPC cells. To further clarify whether eIF4E S209 phosphorylation mediates the YY1 regulation of eIF4E-dependent NPC cell proliferation, we constructed a recombinant eIF4E mutant with S209 substituted by alanine (eIF4E S209A), which cannot be phosphorylation at S209. We used the 5-8F and HNE2 cell lines stably overexpressing YY1, simultaneously overexpressing YY1 and eIF4E(WT), simultaneously overexpressing YY1 and eIF4E (S209A), and the control group. Using the CCK-8 and colony-forming assays, we found that restoring eIF4E S209A expression failed to reverse the inhibitory effect of YY1 overexpression on NPC cell proliferation and colony formation (Figure S2A,B). Consistently, the flow cytometry analysis of cell cycle and apoptosis showed that the eIF4E S209A mutant group did not rescue YY1-induced cell cycle arrest or apoptosis in NPC cells (Figure S2C,D). Additionally, the Western blot analysis revealed that restoring wild-type eIF4E expression at least partially reversed the YY1-mediated regulation of related functional markers (c-PARP, P21, and CDK4). In contrast, the eIF4E S209A mutant failed to rescue the expression of these molecules (Figure S2E).

### 3.4. EIF4E Rescues the Tumor-Suppressive Effect of YY1 on Tumor Growth In Vivo

**Figure 4:**
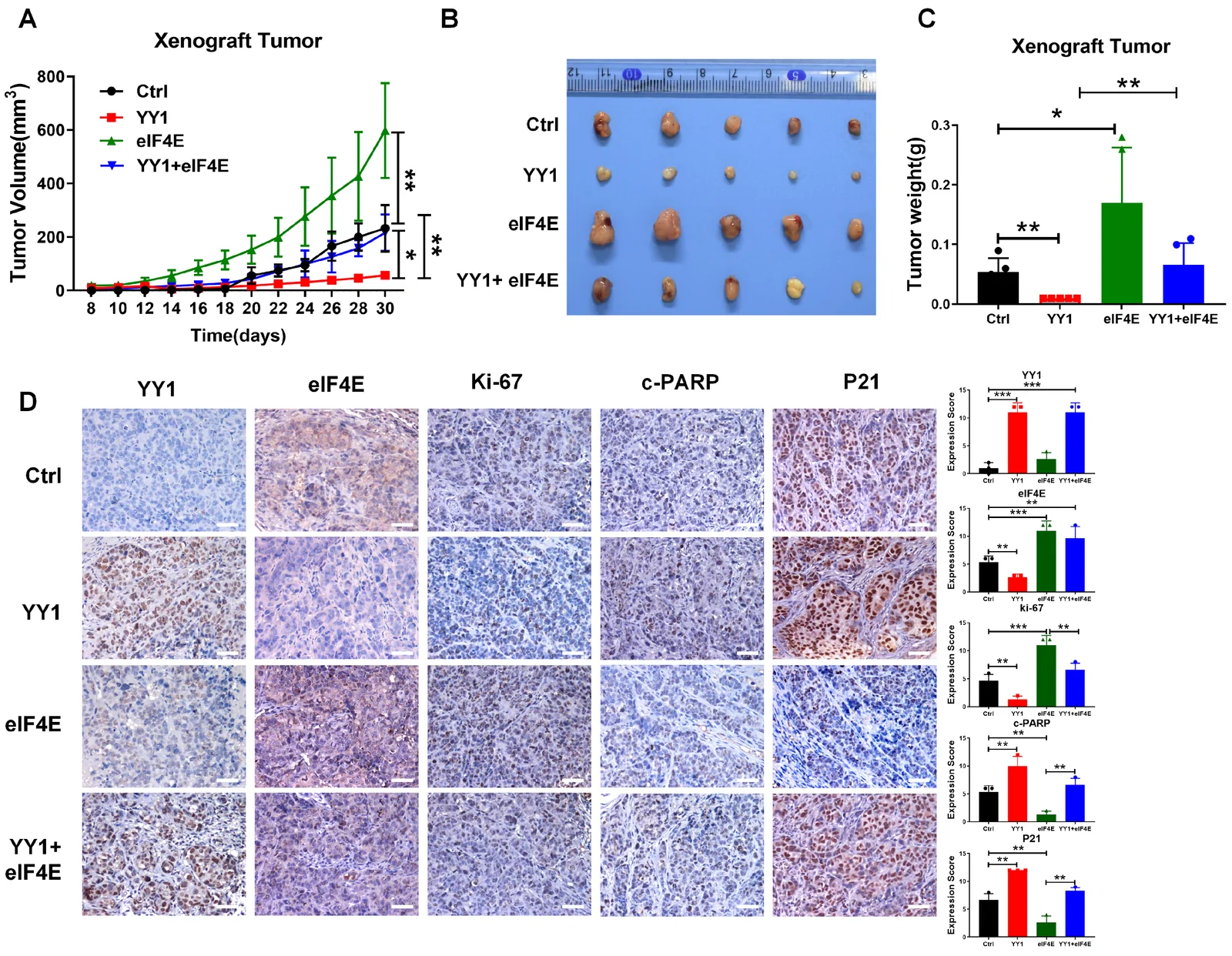
YY1 inhibits tumor growth by down-regulating eIF4E expression in vivo. (**A**) Tumor growth curve (*n* = 5). (**B**) Tumor images of the 5-8F xenograft model in nude mice. (**C**) Tumor weight quantification (*n* = 5). (**D**) Representative IHC images of tumor samples that were stained with YY1, eIF4E, Ki-67, c-PARP, and P21. Three tumors were analyzed per group. Original magnification, 200×; scale bars, 50 μm. Error bars represent the mean ± SD. * *p* < 0.05, ** *p* < 0.01, *** *p* < 0.001.

Given our in vitro findings that eIF4E plays an important role in the YY1 modulation of NPC cell proliferation and apoptosis, we next investigated whether YY1 inhibited tumor growth in vivo by downregulating eIF4E. To this end, a nude mouse xenograft tumor model was established. Specifically, 5-week-old female nude mice were subcutaneously injected into the flank with 5-8F/control cells, 5-8F/YY1 cells, 5-8F/eIF4E cells, or 5-8F/YY1 cells transfected with eIF4E. All mice were euthanized on day 30 post-inoculation (Figure S3), and tumor volumes were measured. As shown in Figure 4A, tumor volumes of mice injected with 5-8F/YY1 cells were significantly reduced compared with those in the negative control group (5-8F/control), while the opposite applied for the 5-8F/eIF4E group. Notably, restoring eIF4E expression in 5-8F/YY1 cells considerably reversed this reduction in tumor volume. Consistent with this, both the weight and growth rate of xenograft tumors of the eIF4E-restored group were significantly increased relative to the 5-8F/YY1 group (Figure 4B,C). To further confirm the effect of eIF4E restoration on tumor proliferation and apoptosis, the percentages and intensity of cells positive for the proliferation markers Ki67, c-PARP, and P21 were analyzed. Ectopic YY1 expression significantly decreased the number of Ki67-positive cells, whereas restoring eIF4E significantly rescued this reduction; these observations were further validated by immunohistochemistry (IHC) staining (Figure 4D). Collectively, these in vivo results demonstrate that eIF4E promotes NPC tumor growth, while YY1 exerts anti-tumor effects, which can be significantly reversed by restoring eIF4E expression.

### 3.5. YY1 Regulates eIF4E-Mediated Translation Function in NPC Cells

**Figure 5:**
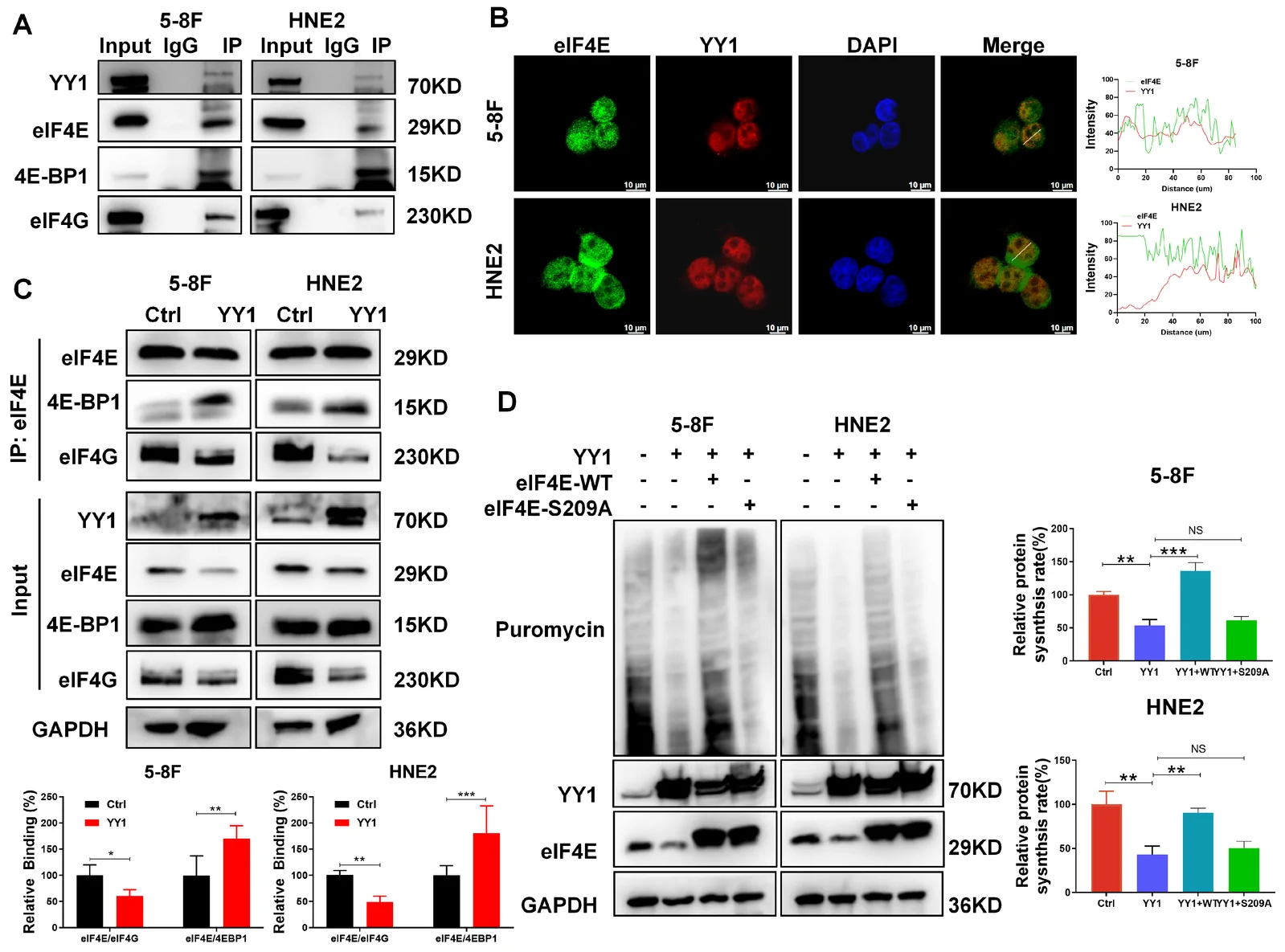
YY1 decreases the interaction between eIF4E and eIF4G in NPC cells. (**A**) Co-IP with anti-YY1 antibody in NPC cells showing interaction between YY1 and endogenous eIF4E, 4E-BP1, and eIF4G, respectively. (**B**) EIF4E and YY1 co-localized in the nucleus of NPC cells. (**C**) Co-IP with an anti-eIF4E antibody in NPC cells showed the effect of YY1 overexpression on the interaction of eIF4E between 4E-BP1 and eIF4G respectively; statistical results were representative of three independent experiments. (**D**) Relative protein synthesis rates were determined by SUnSET assay-Puromycin incorporation. Puromycin incorporation signals were normalized to loading controls, quantified, and expressed as percent relative protein synthesis rates (*n* = 3). Error bars represent the mean ± SD. * *p* < 0.05, ** *p* < 0.01, *** *p* < 0.001; NS, no significant. Original images of the blots are provided in File S1.

4E-BP1 functions as a canonical negative regulator of cap-dependent translation initiation by competitively binding to eIF4E, thereby sterically hindering the recruitment of eIF4G and subsequently disrupting the assembly of the functional eIF4F translation initiation complex. To elucidate the mechanism by which the transcriptional regulator YY1 modulates eIF4E-mediated translational initiation, we systematically investigated the functional and physical interplay among these factors in the context of NPC. The Co-IP assay demonstrated that YY1 physically interacts with endogenous eIF4E, 4E-BP1, and eIF4G in NPC cells, indicating a potential direct regulatory axis (Figure 5A).

The immunofluorescence (IF) assay also indicated that eIF4E and YY1 were co-localized within the nucleus (Figure 5B). These findings suggest that YY1 may affect the translational activity of eIF4E by regulating its translocation from the nucleus to the cytoplasm. Further, we found that YY1 overexpression was accompanied by a marked enhancement in the interaction between eIF4E and 4E-BP1, which reduced the binding between eIF4E and eIF4G, suggesting that YY1 promotes 4E-BP1-mediated sequestration of eIF4E. Consistently, eIF4G expression was decreased upon YY1 overexpression, thereby further inhibiting the formation of the translation initiation complex eIF4F (Figure 5C). Together, these results suggest that YY1 may affect the translational activity of eIF4E. To directly assess translational activity, we performed a puromycin incorporation/SUnSET assay in control, YY1-overexpressing, YY1+eIF4E-WT, and YY1+eIF4E-S209A NPC cells. YY1 overexpression markedly reduced puromycin incorporation, indicating decreased global protein synthesis. Re-expression of eIF4E-WT substantially restored puromycin incorporation, whereas eIF4E-S209A showed a weaker rescue effect, suggesting that eIF4E S209 phosphorylation contributes to YY1-mediated regulation of global translational activity (Figure 5D). However, the specific role of eIF4E S209 phosphorylation in cap-dependent translation requires further experimental validation.

To further investigate the mechanism underlying YY1-mediated regulation of eIF4E activity, we examined the phosphorylation status of 4E-BP1 and related signaling molecules. YY1 overexpression markedly reduced the phosphorylation of 4E-BP1 at Thr37/46 and Ser65 without substantially affecting total 4E-BP1 expression. YY1 overexpression also decreased eIF4E phosphorylation, whereas no corresponding reduction in S6K phosphorylation was observed. These findings suggest that YY1-induced changes in 4E-BP1 phosphorylation are not necessarily accompanied by parallel changes in S6K phosphorylation; however, the involvement of mTORC1 signaling requires further investigation (Figure S4A). Building on these observations, our data further suggest that YY1 regulates multiple aspects of eIF4E-mediated translation initiation, which may in turn contribute to the altered expression of key downstream effector proteins, including Bcl-2, c-Myc, and cyclin D1, all of which are closely associated with cell proliferation and apoptosis (Figure S4B). To further examine the role of eIF4E S209 phosphorylation, we treated YY1-overexpressing and eIF4E-rescued NPC cells with Galeterone to suppress the MNK1/2-eIF4E signaling axis. Western blot analysis showed that Galeterone treatment markedly reduced eIF4E phosphorylation at S209. Notably, Galeterone treatment phenocopied the effect of expressing the eIF4E-S209A mutant in YY1-overexpressing NPC cells (Figure S4C). Consistently, CCK-8 and colony formation assays demonstrated that both Galeterone treatment and expression of the eIF4E-S209A mutant significantly attenuated the malignant phenotypes of NPC cells (Figure S4D,E). These findings suggest that YY1-mediated suppression of eIF4E-dependent translational activity is associated with reduced 4E-BP1 phosphorylation and decreased eIF4E S209 phosphorylation, which may contribute to the inhibition of NPC cell proliferation and malignant progression.

Collectively, these findings uncover a non-canonical model of eIF4E-mediated post-transcriptional regulation: YY1 suppresses translation initiation by enhancing the eIF4E–4E-BP1 interaction and downregulating eIF4G expression, thereby limiting the availability of both eIF4E and eIF4G and ultimately inhibiting eIF4F formation. This study thus provides new insights into the molecular mechanisms underlying translational control in NPC cells.

## 4. Discussion

YY1 is abnormally expressed in a variety of human tumors and exerts dual biological functions as a tumor suppressor gene or oncogene by regulating different target genes or signaling pathways. In the vast majority of tumors, such as hepatocellular carcinoma, colorectal cancer, and cervical cancer, YY1 expression in tumor biopsy tissues is significantly increased compared with the corresponding adjacent or normal tissues. YY1 overexpression promotes tumor proliferation, invasion, and metastasis by regulating different signaling pathways or key molecules; therefore, YY1 functions as an oncogene [28,29,30]. However, in pancreatic cancer biopsy tissues, high YY1 expression has been positively correlated with the efficacy of chemotherapy and survival rate and has been found to promote cell apoptosis by increasing Bax expression and activation [31,32]. In breast cancer cells, YY1, as a transcription factor, directly binds to the promoter region of BRCA1, a breast cancer susceptibility gene, positively regulating its transcriptional activity and expression; YY1 overexpression even prevented the proliferation, clone formation, and tumorigenesis of breast cancer cells in nude mice [33]. Therefore, YY1 plays the role of a suppressor gene in tumors such as pancreatic cancer and breast cancer. In recent years, an increasing number of genes, such as Claudin-1 [34], KLF4 [35], ATF-2 [36] and TGF-β [37], have been found to have dual functions in tumors. For example, Claudin-1 was highly expressed in tumors such as colon cancer, oral squamous cell carcinoma, some subtypes of breast cancer, and melanoma and promoted tumor growth and metastasis as an oncogene; however, it was lowly expressed in tumors such as hepatocellular carcinoma and lung adenocarcinoma and inhibited tumor invasion and metastasis as a tumor suppressor gene [34]. In conclusion, YY1 is an important transcription factor that exerts the dual functions of tumor oncogene or tumor suppressor gene in different tumors. The specific function depends on the type of tumor or cell and the stage of tumor development.

eIF4E plays a crucial role in the initiation and progression of a variety of tumors. In HNSCC, eIF4E was suggested to be a more significant prognostic indicator than p53 [38]. In addition, eIF4E and Cyclin D1 expression were increased in HNSCC, with both genes stimulating cell cycle progression and transforming squamous epithelial cells [39]. Franklin et al. showed that eIF4E was overexpressed in the tumor in patients and that its overexpression at the incisal edge increased the probability of larynx and hypopharynx cancer recurrence [40]. Liang et al. confirmed that eIF4E was highly expressed in laryngeal squamous cell carcinoma but not in polyp tissue. eIF4E overexpression could regulate bFGF, with both playing an important synergistic role in the occurrence, invasion, and metastasis of laryngeal squamous cell carcinoma [41]. According to the report by Dong et al., eIF4E expression in serum was significantly higher in NSCLC patients than that in the control group, and eIF4E was an independent prognostic factor for shortened overall survival and progression-free survival [42]. As Qi et al. showed, eIF4E was highly expressed in multiple lung cancer cell lines, and siRNA-eIF4E could significantly inhibit lung cancer cell proliferation [43]. Li et al. found that eIF4E was positively regulated by RPUSD1 to activate the PI3K/AKT signaling pathway in non-small cell lung cancer, thereby promoting tumor cell proliferation and invasion [44]. Moreover, eIF4E is dysregulated in breast cancers, in which it is used as a prognostic indicator [45]. Kerekatte et al. showed that eIF4E was overexpressed in breast cancer but not in normal breast tissue or benign breast lesions (hyperplasia or inflammation) [46]. Gao et al. found that eIF4E was upregulated in CRC and that its expression frequency (EF) in cancer tissues was higher than that in normal adjacent tissues [47]. In addition, eIF4E mediated the biogenesis of circPHF14, which promoted the growth and metastasis of pancreatic ductal adenocarcinoma via the Wnt/β-catenin pathway [48]. In this study, we confirmed that eIF4E promoted NPC growth, whereas YY1 exerted tumor-suppressive effects partly through negative regulation of eIF4E.

S209 phosphorylation is the best-characterized post-translational modification of eIF4E and plays an important role in regulating its oncogenic activity [49]. The phosphorylation of eIF4E could stimulate the translation of tumor-promoting mRNAs, thus enhancing its carcinogenic characteristics [50]. Yoshizawa et al. showed that the overexpression rate of p-eIF4E in 300 samples of NSCLC was 39.9%, indicating that the activity of eIF4E in NSCLC was increased [51]. Studies have shown that upregulated PRSS23 further promotes the transcriptional activity of c-myc by binding to eIF4E S209, thus promoting the occurrence and development of gastric cancer cells [52]. SENP3 desumoylates RACK1 and increases its stability and interaction with PKCβII, thereby promoting eIF4E phosphorylation and CCL20 translation, which prompts the infiltration of hepatocellular carcinoma-associated macrophages (TAMs) while reducing cytotoxic T cells to facilitate immune evasion [53]. In HCT116 cells carrying the eIF4E-S209A/+ heterozygous knock-in, total eIF4E levels, cap binding, and global translation were largely unaffected, whereas cell growth in spheroids and mice and CRC organoid growth were markedly reduced, likely due to strong inhibition of Myc and ATF4 translation, the ISR-dependent glutamine metabolic signature, and AKT activation [54]. This study further demonstrates that restoring expression of eIF4E-WT, but not eIF4E-S209A, partially reverses the inhibitory effects of YY1 on NPC cell proliferation, cell-cycle progression, and apoptosis resistance, supporting a functional role of eIF4E phosphorylation at S209 in YY1-mediated regulation of these phenotypes.

eIF4E is a key rate-limiting factor in cap-dependent translation initiation and is particularly important for the translation of mRNAs with highly structured 5′ untranslated regions [55]. To ensure that protein synthesis occurs on demand, cells have evolved multi-layered regulatory mechanisms to govern eIF4E activity, which include transcriptional regulation. Studies have shown that oncogenes such as MYC can activate the transcription of the eIF4E gene, thereby subsequently increasing eIF4E mRNA and protein levels [56]. Moreover, it has been reported that expression of eIF4E relative to 4E-BP1 is a more precise predictor of HNSCC and its progression [57]. Our previous study confirmed that YY1 negatively regulates the transcriptional activity and expression of eIF4E by targeting its promoter region. Additionally, 4E-BP1 inhibits translation initiation by competing with eIF4G for binding to eIF4E, thereby limiting eIF4F complex assembly [58].

Although eIF4E is predominantly cytoplasmic, we observed both cytoplasmic and nuclear localization of eIF4E and partial nuclear co-localization with the transcription factor YY1 in NPC cells. These findings suggest that YY1 may regulate eIF4E at multiple levels. In addition to suppressing eIF4E transcription, YY1 overexpression reduced 4E-BP1 phosphorylation at Thr37/46 and Ser65, which was accompanied by enhanced eIF4E-4E-BP1 association and reduced eIF4E-eIF4G interaction. These changes are consistent with reduced eIF4F complex formation and attenuated eIF4E-dependent translational activity. However, whether these effects specifically reflect alterations in cap-dependent translation requires further experimental validation. Importantly, although the Co-IP results support changes in the assembly of eIF4E-containing complexes, they do not provide direct evidence for competitive binding between eIF4E and 4E-BP1 or eIF4G. Additional experiments will be required to confirm this mechanism.

Moreover, the mechanisms underlying NPC development remain incompletely understood. Increasing evidence suggests that cancer should not be regarded solely as a genetic or molecular disease, but rather as a complex system shaped by interactions among tumor cells, immune and stromal components, metabolic states, and other features of the tumor microenvironment [59]. From this perspective, the YY1-eIF4E axis identified in the present study may also interact with broader tumor ecological factors. Future studies should further investigate these interactions to better understand NPC progression and therapeutic response.

## 5. Conclusions

Our findings demonstrated that eIF4E promotes NPC cell proliferation, facilitates G1/S transition, and confers resistance to apoptosis. Moreover, restoration of eIF4E expression at least partially reversed the tumor-suppressive effects of YY1 on NPC cell proliferation and apoptosis both in vitro and in vivo. Importantly, YY1 overexpression was associated with enhanced eIF4E−4E-BP1 association and reduced eIF4E-eIF4G interaction, changes consistent with reduced eIF4F complex formation and attenuated eIF4E-dependent translation initiation. Collectively, these findings suggest that YY1 suppresses NPC growth by negatively regulating both eIF4E expression and its translational function.

## Data Availability

The datasets generated during and analyzed during the current study are available from the corresponding author upon reasonable request.

## Supplementary Materials

The following supporting information can be downloaded at: https://www.mdpi.com/article/10.3390/cancers18203257/s1. Figure S1: EIF4E is highly expressed in HNSC. Figure S2: The promoting effect on the proliferation of NPC cells depends on the activation of eIF4E S209. Figure S3: YY1 inhibits tumor growth by down-regulating eIF4E expression in vivo. Figure S4: YY1 suppresses NPC cell growth by inhibiting eIF4E phosphorylation and translation activity; File S1: Original Images for Blots.

## Abbreviations

The following abbreviations are used in this manuscript:

NPC	Nasopharyngeal carcinoma
YY1	Yinyang-1
eIF4E	Eukaryotic Translation Initiation Factor 4E
eIF4G	Eukaryotic Translation Initiation Factor 4G
eIF4F	Eukaryotic initiation factor 4F
4E-BP1	EIF4E-Binding Protein 1
CCK-8	Cell Counting Kit-8
IF	Immunofluorescence
Co-IP	Co-immunoprecipitation
IHC	Immunohistochemistry
PBS	Phosphate-buffered saline
PI	Propidium iodide
SDS-PAGE	Sodium dodecyl sulfate-polyacrylamide gel electrophoresis
cDNA	Complementary DNA
CDS	Coding sequence
