# Supplementary material for: YY1 Suppresses Tumor Growth by Negatively Regulating the Expression and Translation Activity of eIF4E in Nasopharyngeal Carcinoma
Source: Cancers (Basel). 2026 Oct 9;18(20):3257. doi: 10.3390/cancers18203257 (PMC13702381; doi:10.3390/cancers18203257)
Supplement: Supplementary file 1 [file cancers-18-03257-s001.zip › cancers-4432541-Supplementary File S1-original blot.pdf]

Original Images for Blots

YY1 Suppresses Tumor Growth by Negatively Regulating the Expression and Translation Activity of eIF4E in Nasopharyngeal Carcinoma

Figure 1A

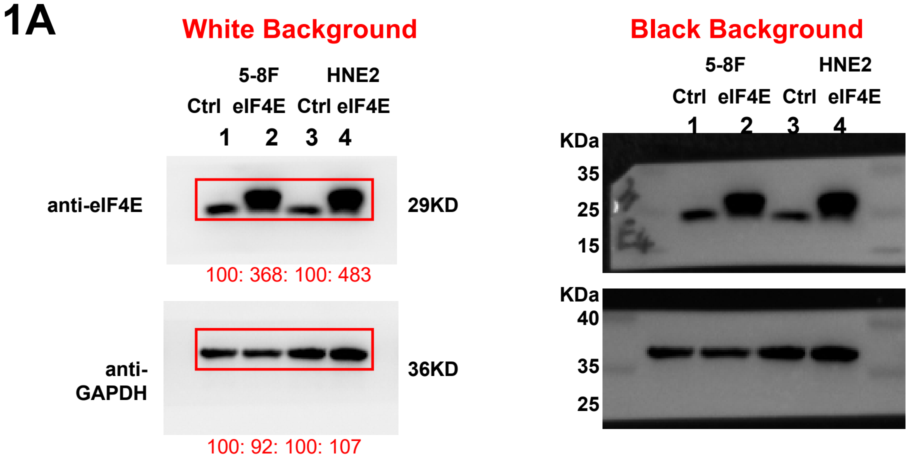

Figure 1F

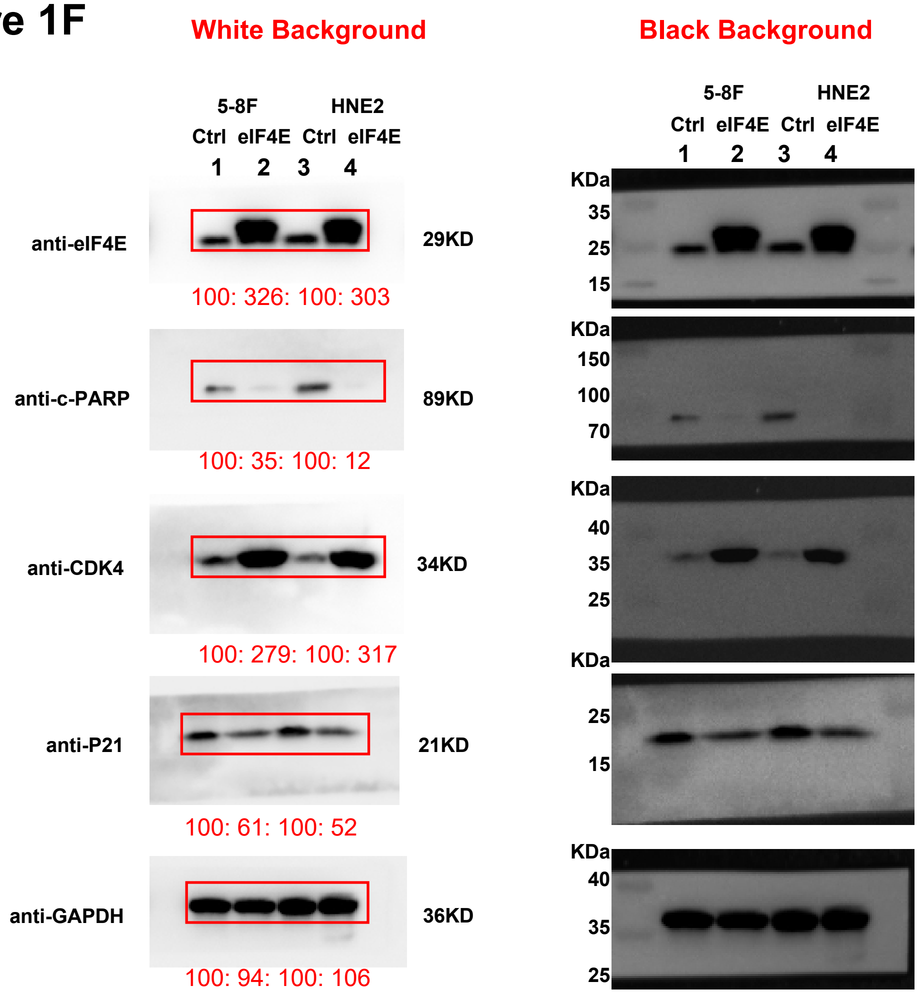

Figure 2A

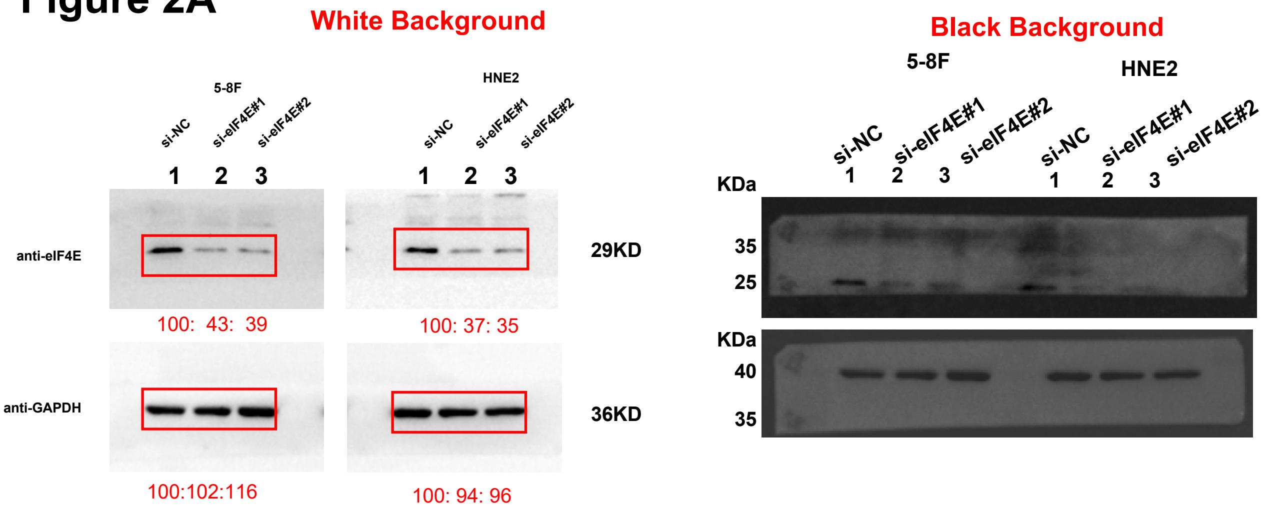

Figure 2F

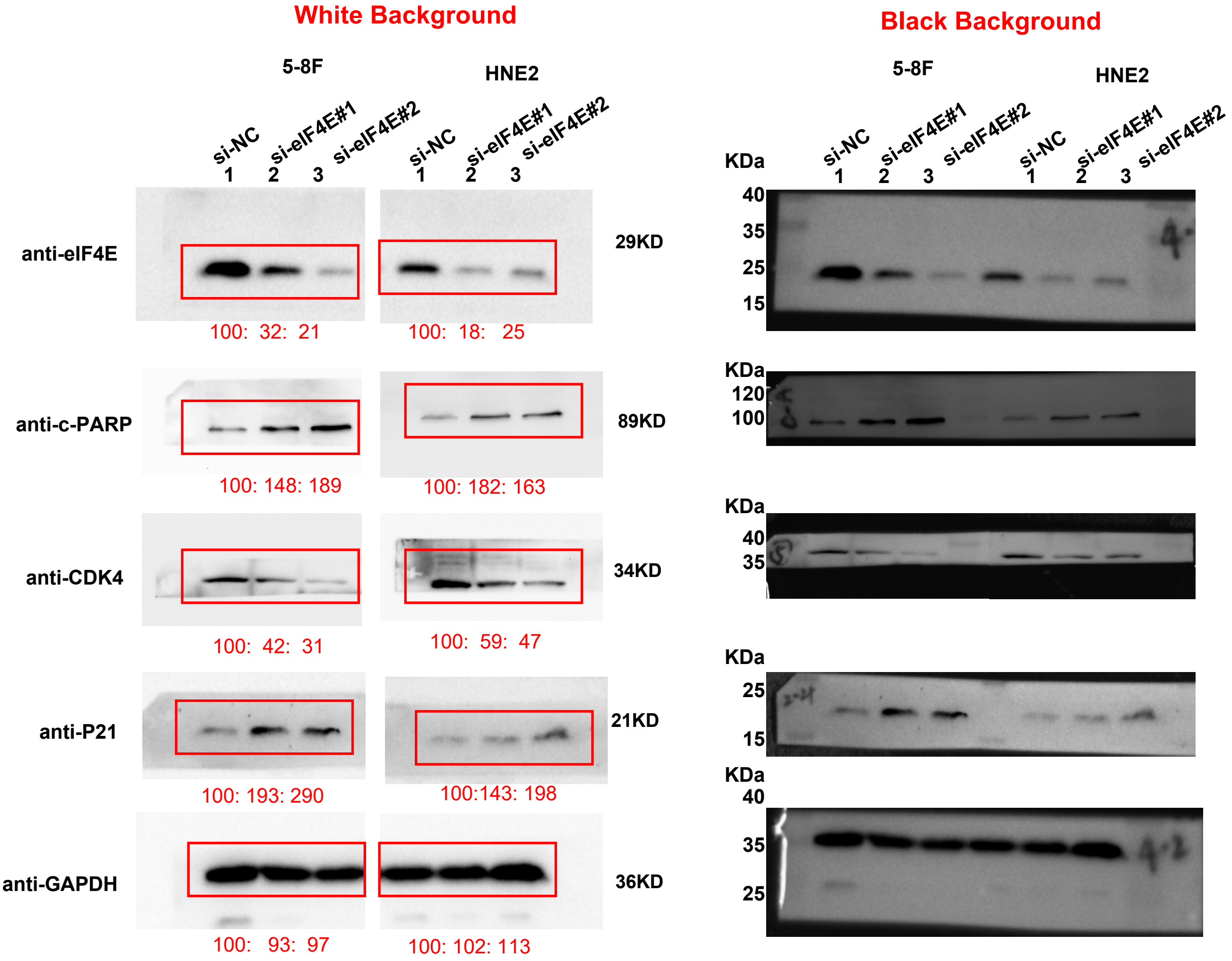

Figure 3A

White Background

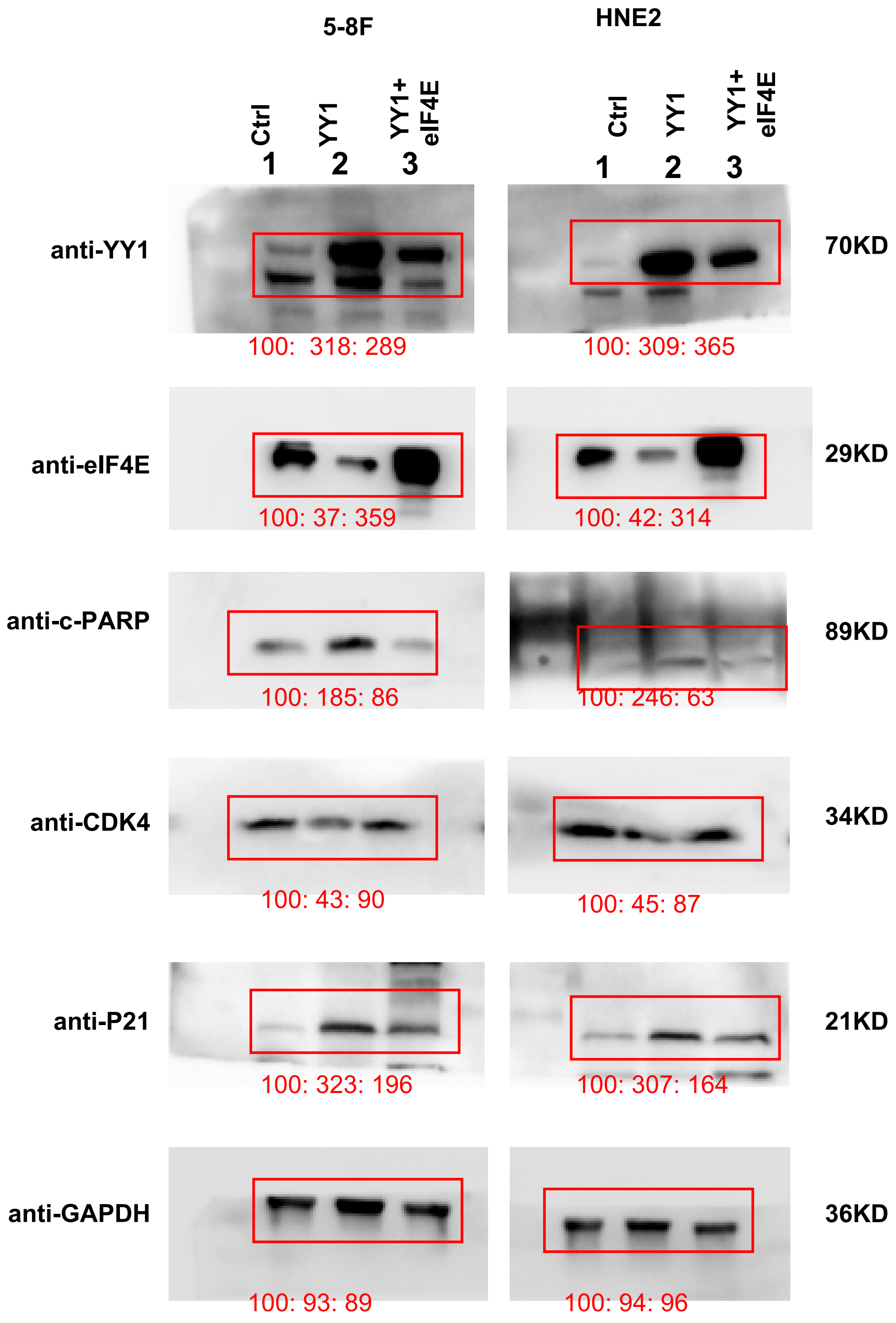

Figure 3A

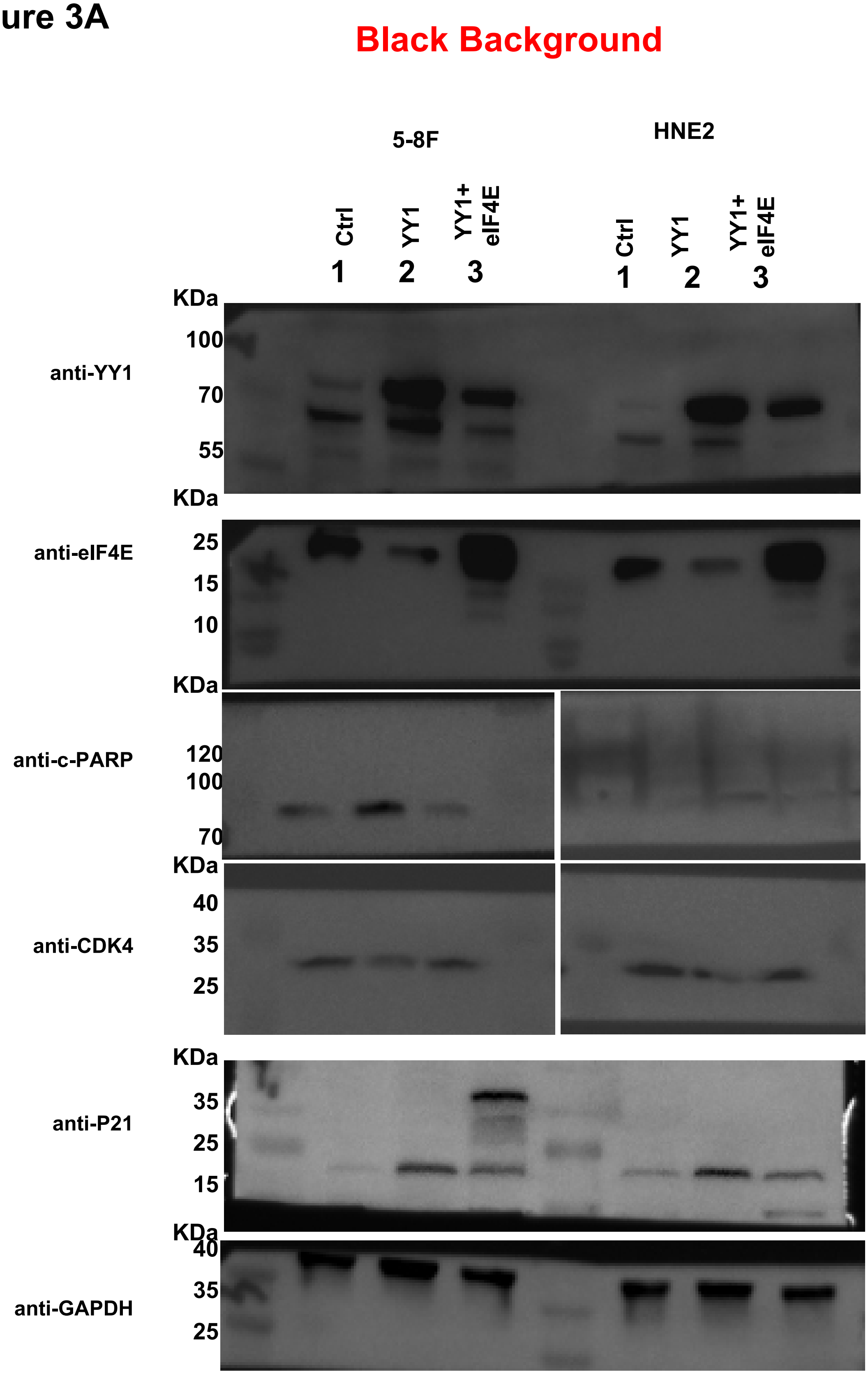
Black Background
5-8F
HNE2
Ctrl
YY1
YY1+ eIF4E
1
2
3
KDa
100
70
55
25
15
10
120
40
35
anti-YY1
anti-eIF4E
anti-c-PARP
anti-CDK4
anti-P21
anti-GAPDH

Figure 5A

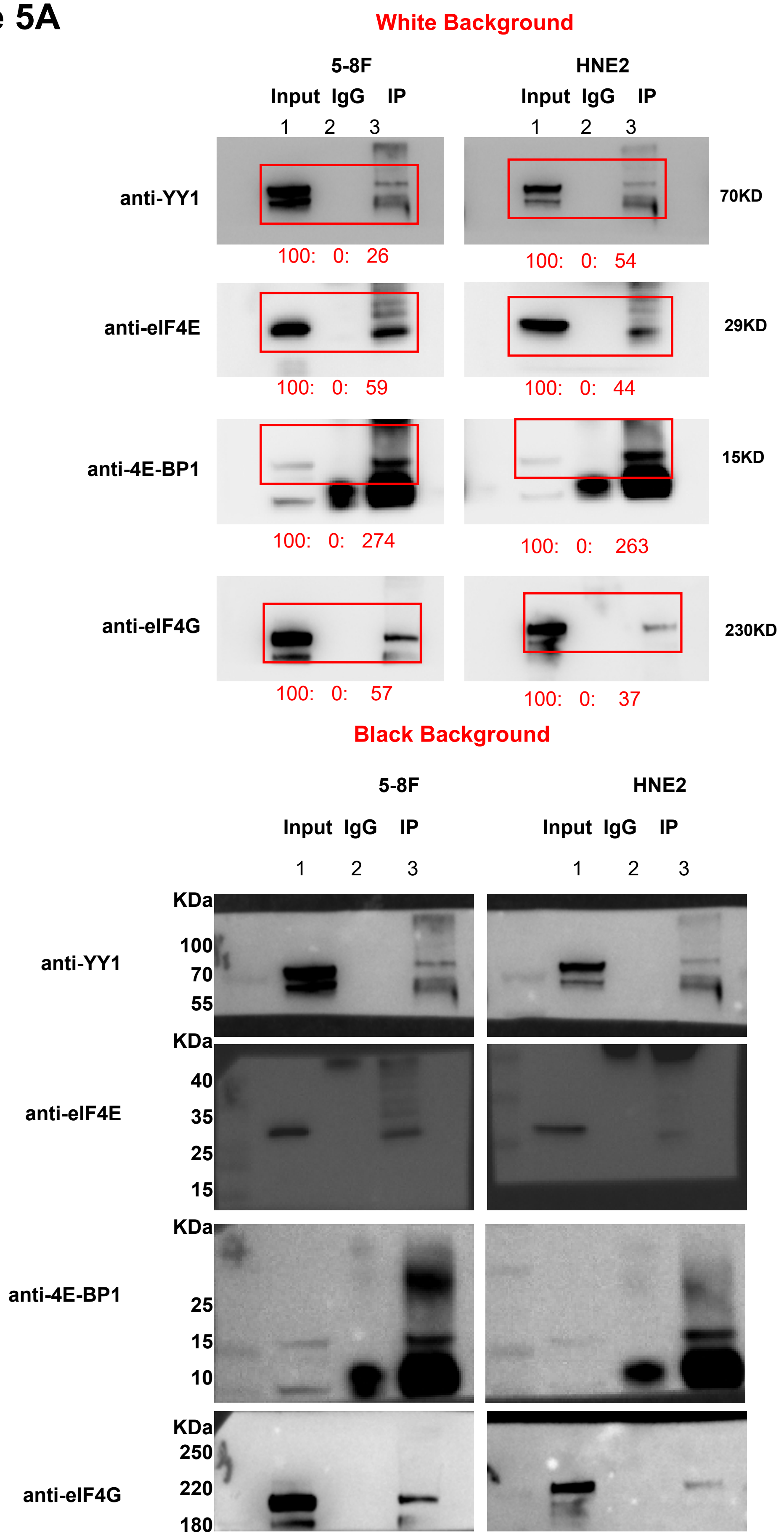

White Background
5-8F
HNE2
Input IgG IP
1 2 3
anti-YY1
70KD
100: 0: 26
100: 0: 54
anti-eIF4E
29KD
100: 0: 59
100: 0: 44
anti-4E-BP1
15KD
100: 0: 274
100: 0: 263
anti-eIF4G
230KD
100: 0: 57
100: 0: 37
Black Background
KDa
100
70
55
40
35
25
15
10
250
220
180

Figure 5C

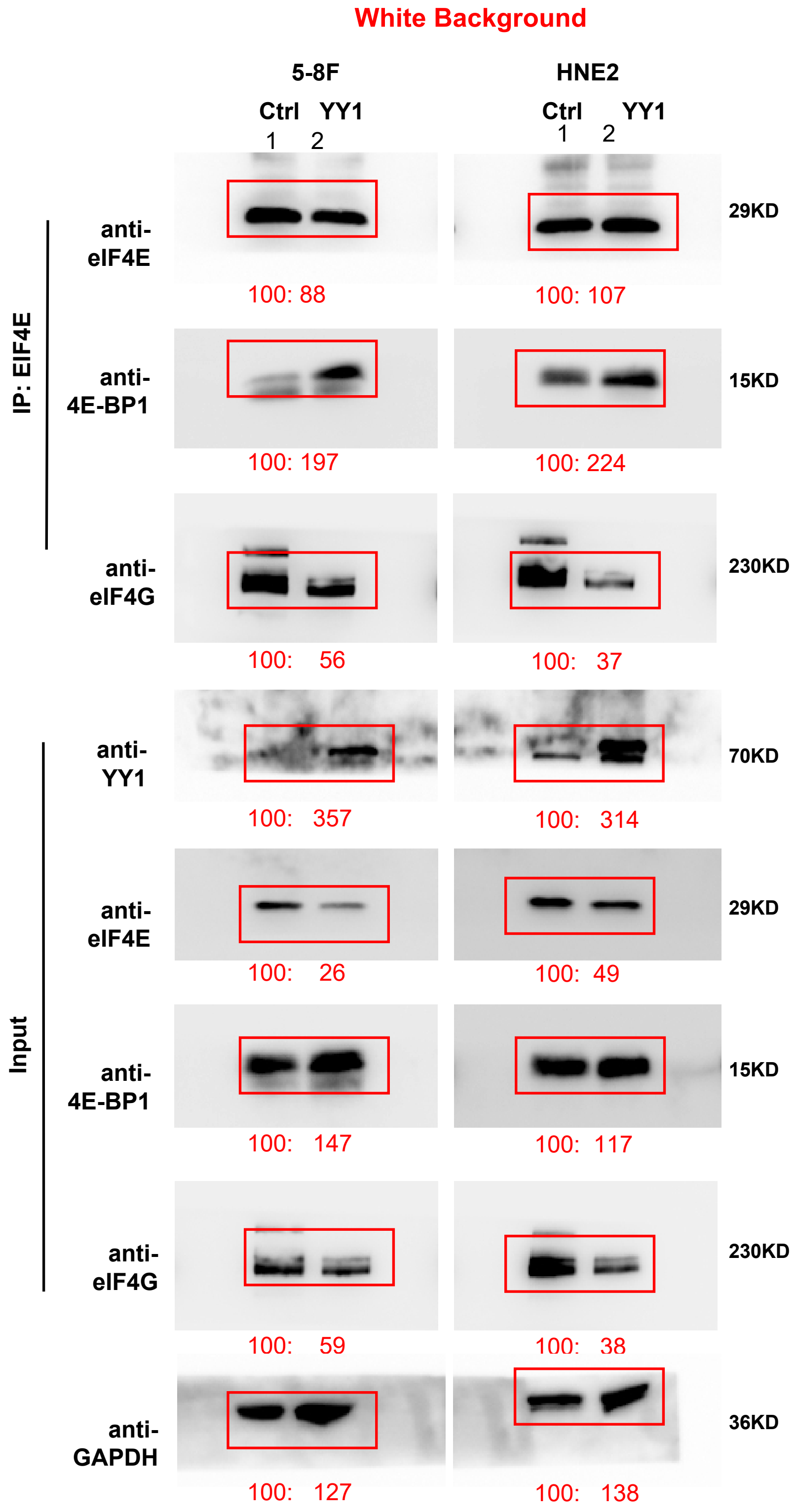
White Background
5-8F
HNE2
Ctrl YY1
1 2
Ctrl YY1
1 2
IP: EIF4E
anti-eIF4E
100: 88
100: 107
29KD
anti-4E-BP1
100: 197
100: 224
15KD
anti-eIF4G
100: 56
100: 37
230KD
Input
anti-YY1
100: 357
100: 314
70KD
anti-eIF4E
100: 26
100: 49
29KD
anti-4E-BP1
100: 147
100: 117
15KD
anti-eIF4G
100: 59
100: 38
230KD
anti-GAPDH
100: 127
100: 138
36KD

Figure 5C

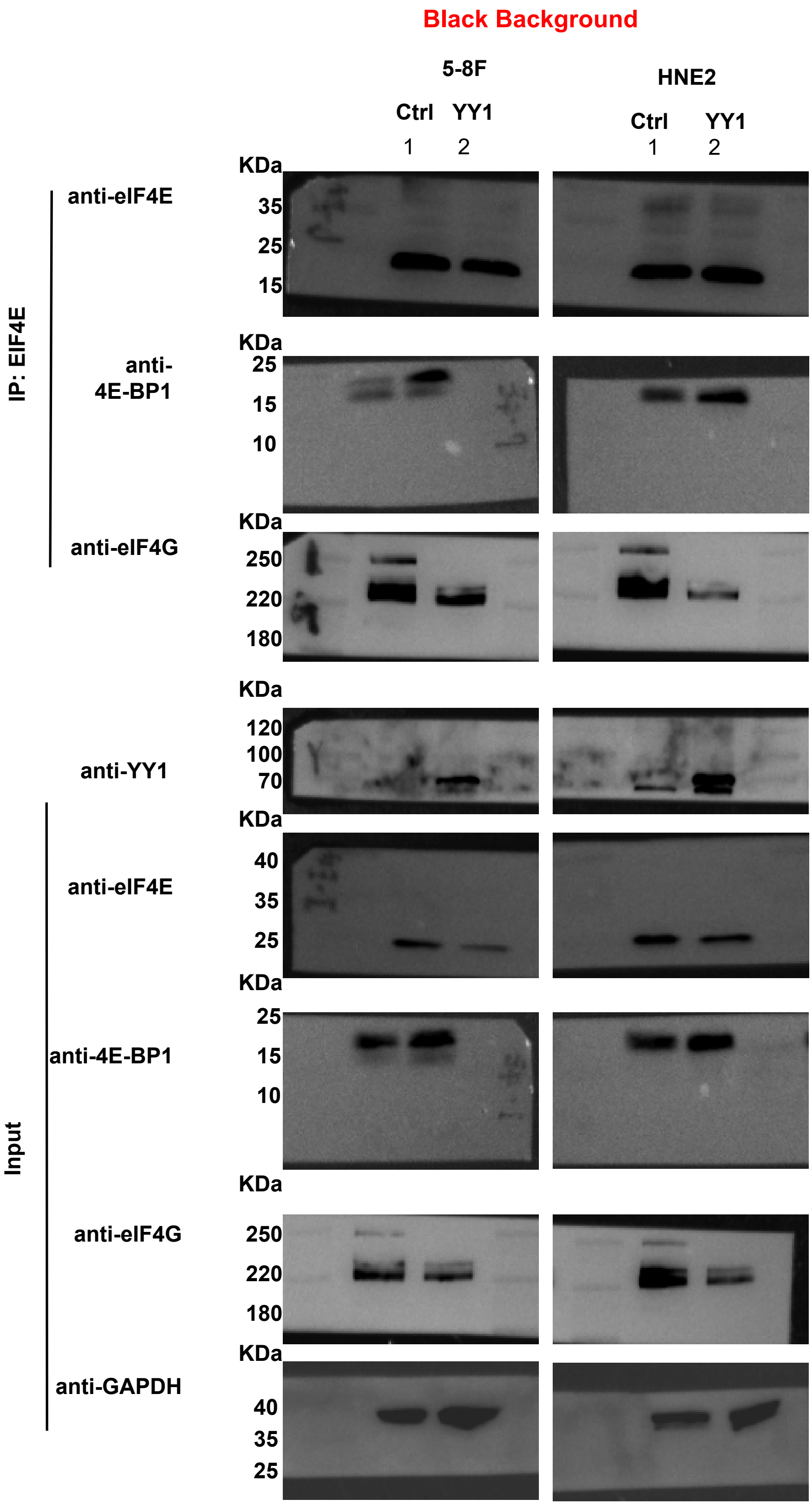

Black Background
5-8F
HNE2
Ctrl YY1
1 2
Ctrl YY1
1 2
IP: EIF4E
Input
anti-eIF4E
anti-4E-BP1
anti-eIF4G
anti-YY1
anti-eIF4E
anti-4E-BP1
anti-eIF4G
anti-GAPDH
KDa
35
25
15
KDa
25
15
10
KDa
250
220
180
KDa
120
100
70
KDa
40
35
25
KDa
25
15
10
KDa
250
220
180
KDa
40
35
25

Figure 5D

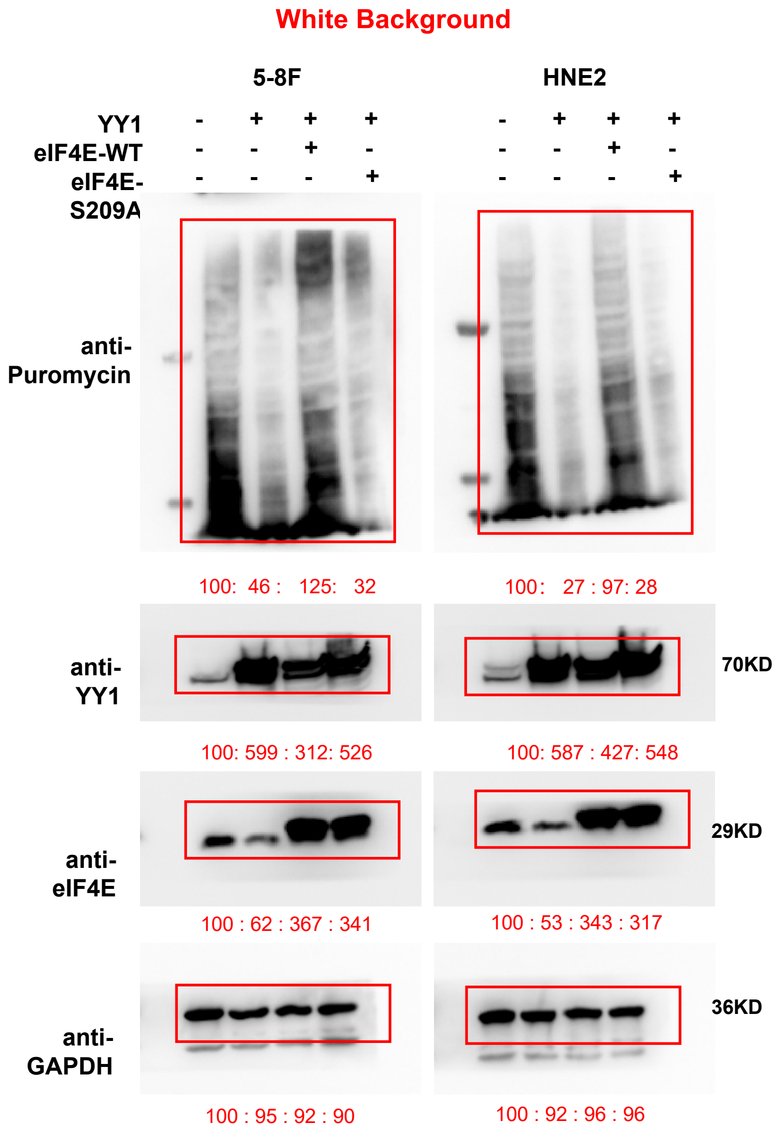
White Background
5-8F
HNE2
YY1 - + + + - + + +
eIF4E-WT - - + - - - + -
eIF4E-S209A - - - + - - - +
anti-Puromycin
100: 46 : 125: 32
100: 27 : 97: 28
anti-YY1
70KD
100: 599 : 312: 526
100: 587 : 427: 548
anti-eIF4E
29KD
100 : 62 : 367 : 341
100 : 53 : 343 : 317
anti-GAPDH
36KD
100 : 95 : 92 : 90
100 : 92 : 96 : 96

Figure 5D

Black Background

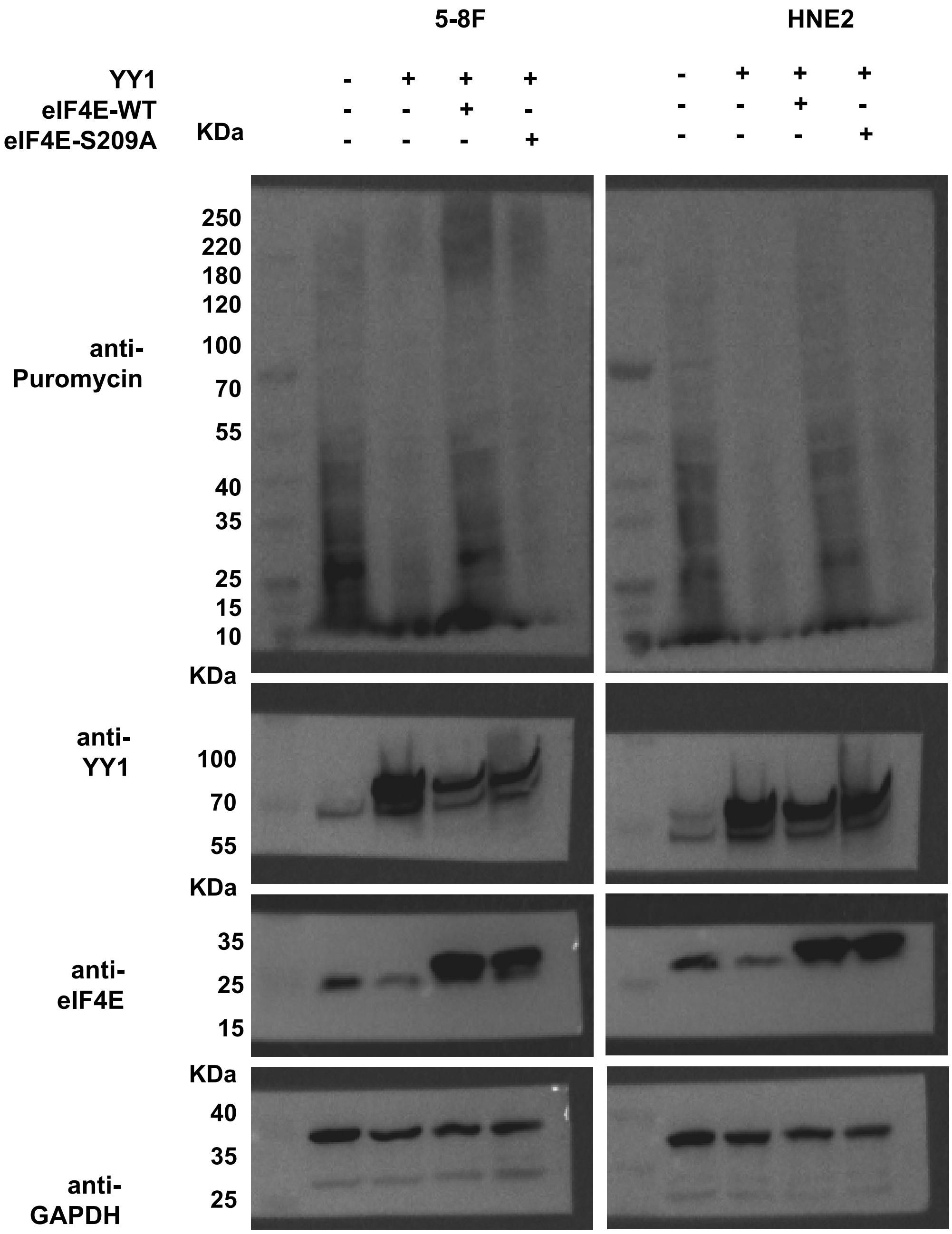

Figure S2C

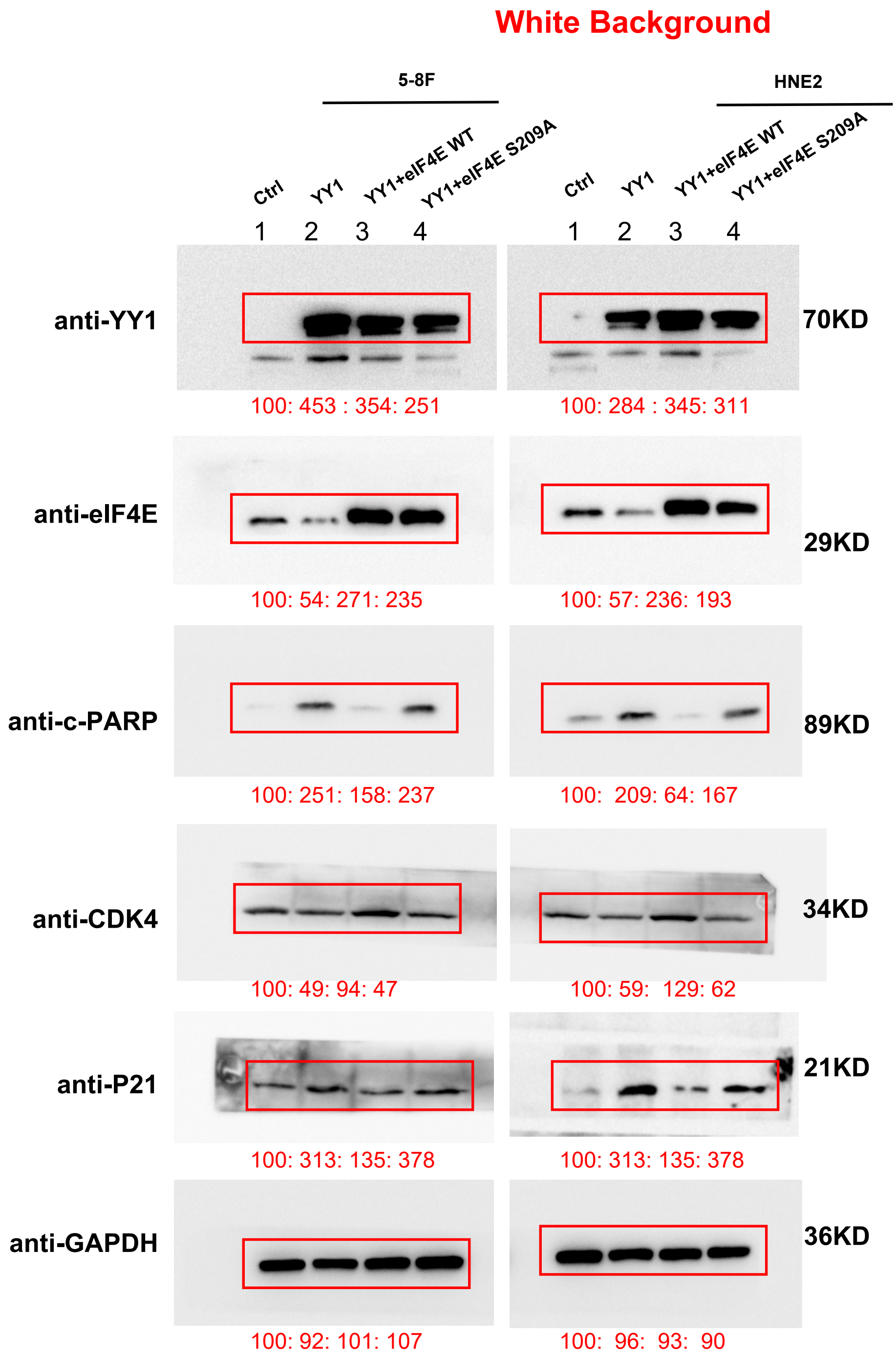

White Background
5-8F
HNE2
Ctrl
YY1
YY1+eIF4E WT
YY1+eIF4E S209A
1 2 3 4
1 2 3 4
anti-YY1
70KD
100: 453 : 354: 251
100: 284 : 345: 311
anti-eIF4E
29KD
100: 54: 271: 235
100: 57: 236: 193
anti-c-PARP
89KD
100: 251: 158: 237
100: 209: 64: 167
anti-CDK4
34KD
100: 49: 94: 47
100: 59: 129: 62
anti-P21
21KD
100: 313: 135: 378
100: 313: 135: 378
anti-GAPDH
36KD
100: 92: 101: 107
100: 96: 93: 90

Figure S2C

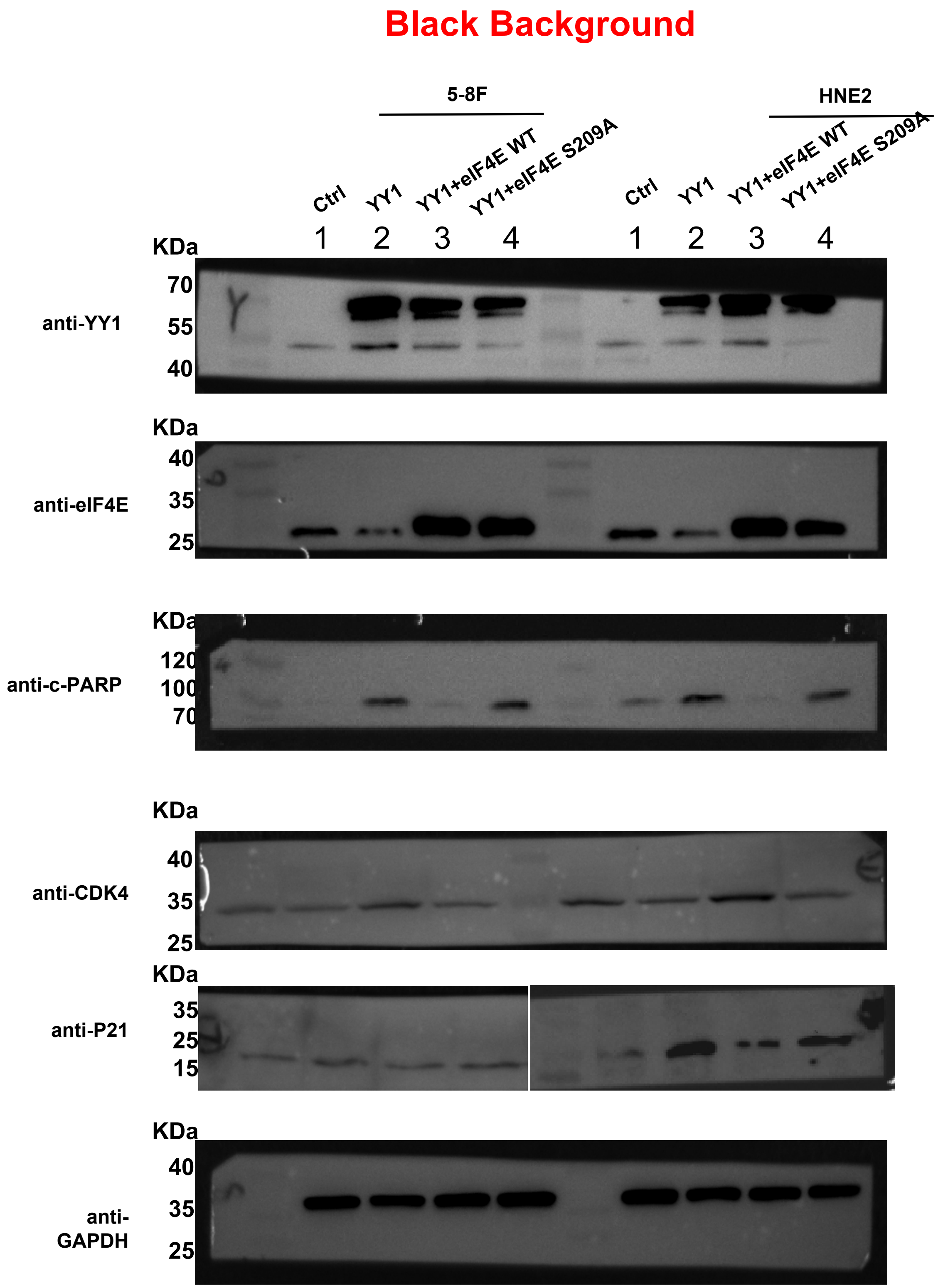
Black Background
5-8F
HNE2
Ctrl
YY1
YY1+eIF4E WT
YY1+eIF4E S209A
1 2 3 4
1 2 3 4
KDa
70
55
40
anti-YY1
anti-eIF4E
35
25
anti-c-PARP
120
100
70
anti-CDK4
anti-P21
15
anti-GAPDH

Figure S4A

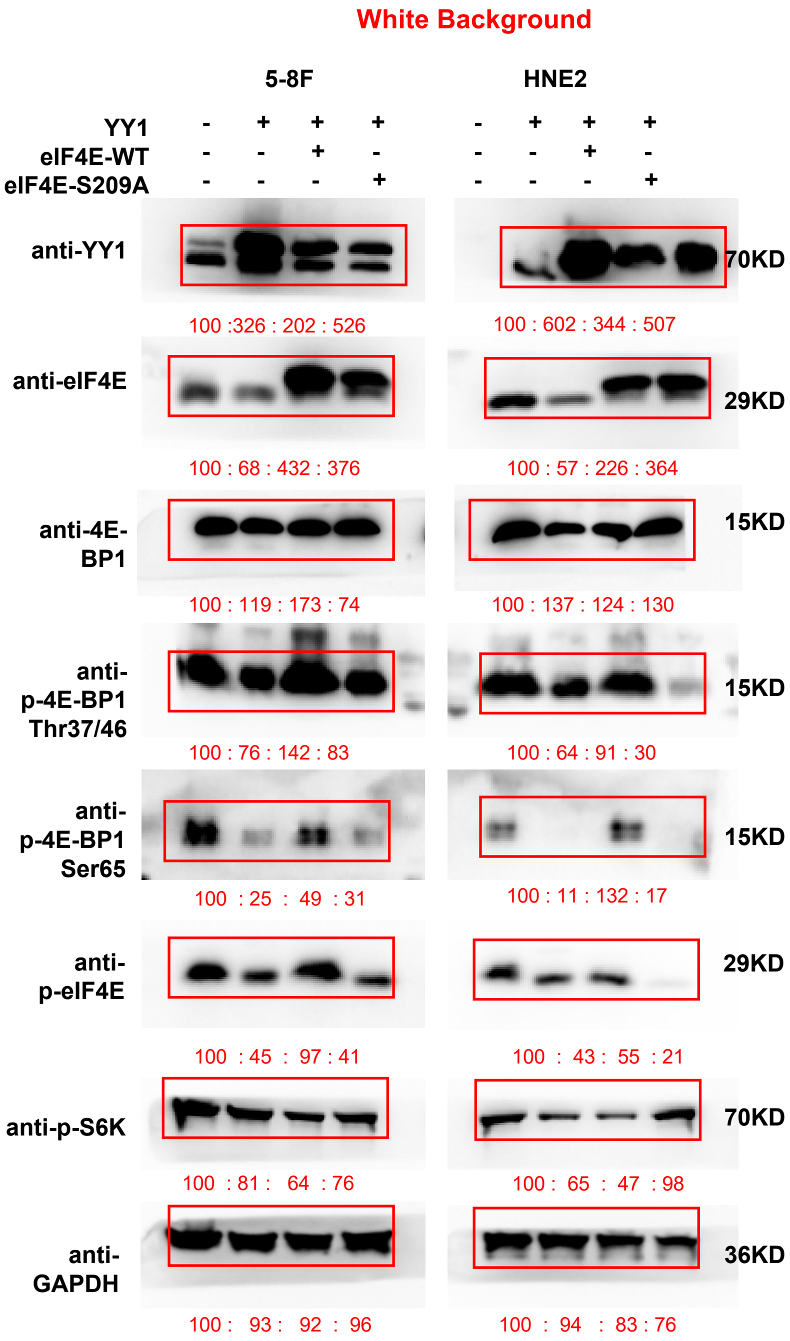
White Background
5-8F
HNE2
YY1 - + + + - + + +
eIF4E-WT - - + - - - + -
eIF4E-S209A - - - + - - - +
anti-YY1
70KD
100 :326 : 202 : 526
100 : 602 : 344 : 507
anti-eIF4E
29KD
100 : 68 : 432 : 376
100 : 57 : 226 : 364
anti-4E-BP1
15KD
100 : 119 : 173 : 74
100 : 137 : 124 : 130
anti-p-4E-BP1 Thr37/46
15KD
100 : 76 : 142 : 83
100 : 64 : 91 : 30
anti-p-4E-BP1 Ser65
15KD
100 : 25 : 49 : 31
100 : 11 : 132 : 17
anti-p-eIF4E
29KD
100 : 45 : 97 : 41
100 : 43 : 55 : 21
anti-p-S6K
70KD
100 : 81 : 64 : 76
100 : 65 : 47 : 98
anti-GAPDH
36KD
100 : 93 : 92 : 96
100 : 94 : 83 : 76

Figure S4A

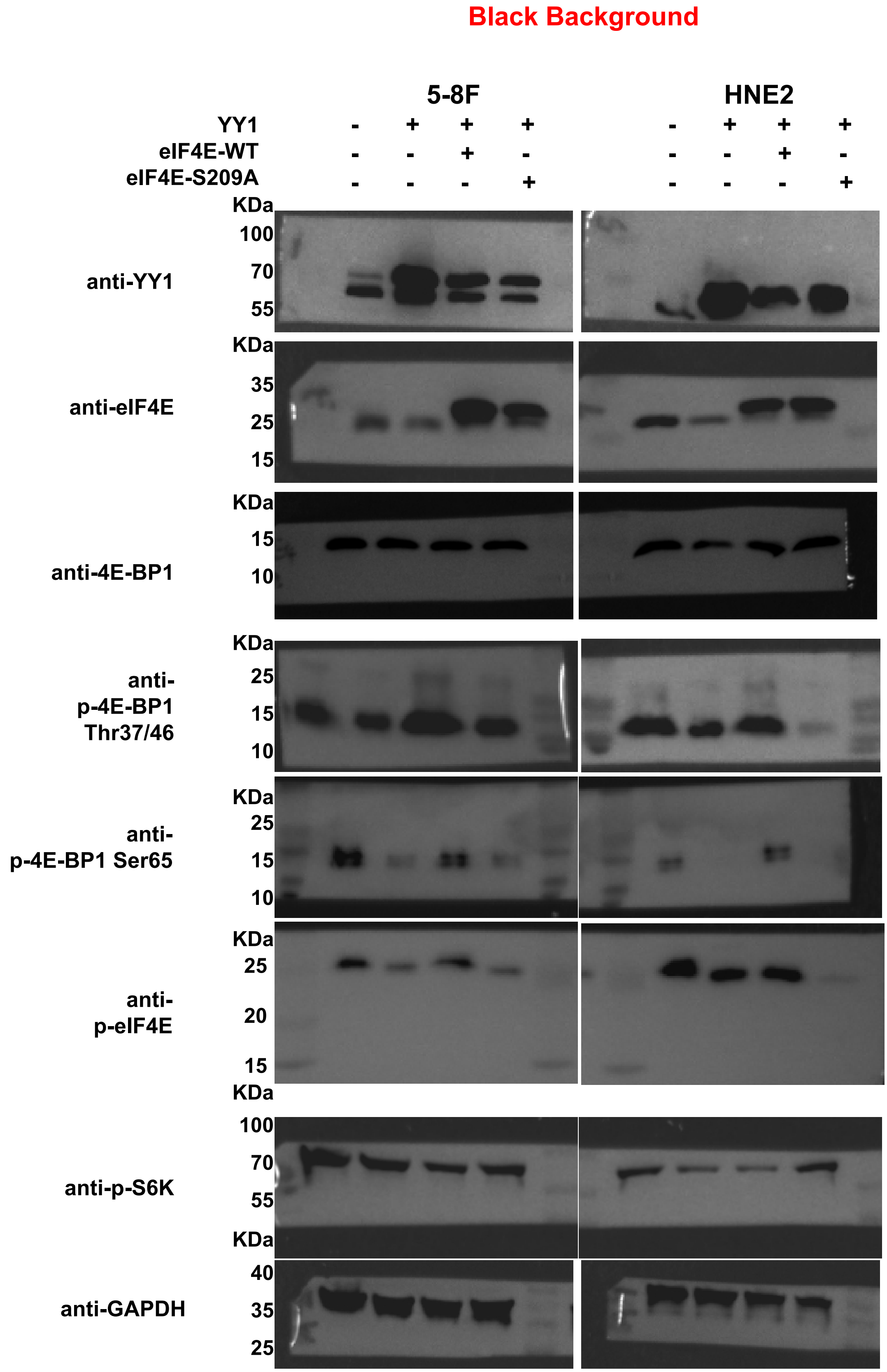
Black Background
5-8F
HNE2
YY1 - + + + - + + +
eIF4E-WT - - + - - - + -
eIF4E-S209A - - - + - - - +
KDa
100
70
55
anti-YY1
KDa
35
25
15
anti-eIF4E
KDa
15
10
anti-4E-BP1
KDa
25
15
10
anti-p-4E-BP1 Thr37/46
KDa
25
15
10
anti-p-4E-BP1 Ser65
KDa
25
20
15
anti-p-eIF4E
KDa
100
70
55
anti-p-S6K
KDa
40
35
25
anti-GAPDH

Figure S4B

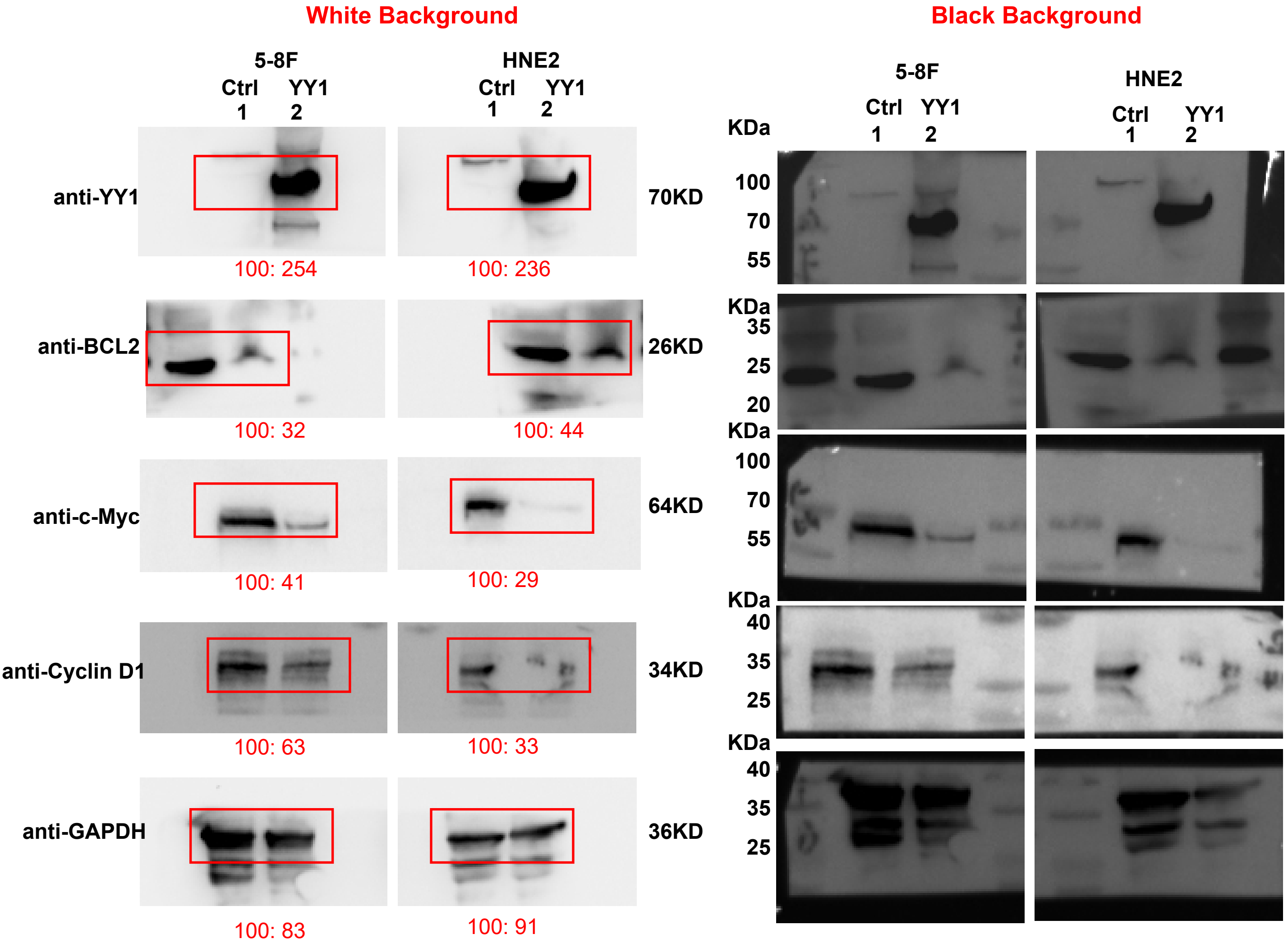
White Background
Black Background
5-8F
HNE2
Ctrl YY1
1 2
anti-YY1
anti-BCL2
anti-c-Myc
anti-Cyclin D1
anti-GAPDH
70KD
26KD
64KD
34KD
36KD
100: 254
100: 236
100: 32
100: 44
100: 41
100: 29
100: 63
100: 33
100: 83
100: 91
KDa
100
70
55
35
25
20
40

Figure S4C

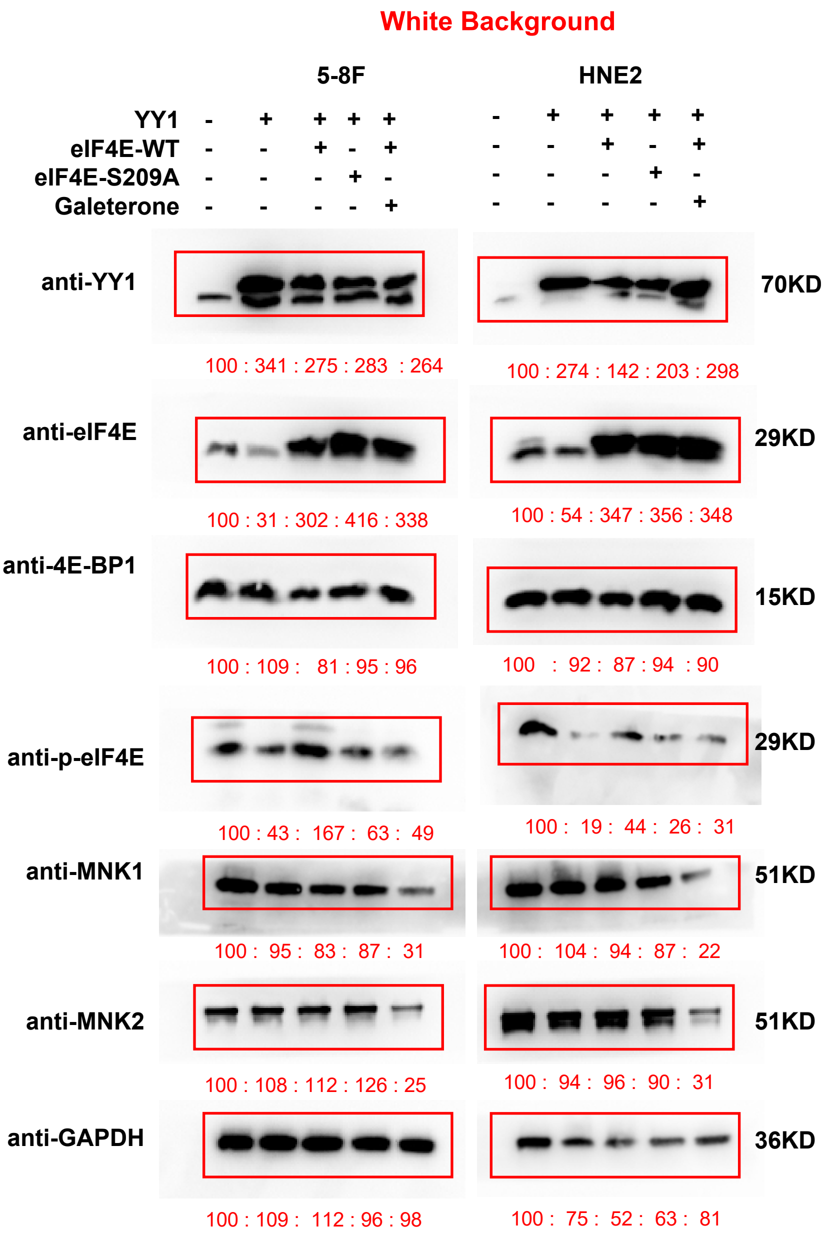
White Background
5-8F
HNE2
YY1
eIF4E-WT
eIF4E-S209A
Galeterone
anti-YY1
70KD
100 : 341 : 275 : 283 : 264
100 : 274 : 142 : 203 : 298
anti-eIF4E
29KD
100 : 31 : 302 : 416 : 338
100 : 54 : 347 : 356 : 348
anti-4E-BP1
15KD
100 : 109 : 81 : 95 : 96
100 : 92 : 87 : 94 : 90
anti-p-eIF4E
29KD
100 : 43 : 167 : 63 : 49
100 : 19 : 44 : 26 : 31
anti-MNK1
51KD
100 : 95 : 83 : 87 : 31
100 : 104 : 94 : 87 : 22
anti-MNK2
51KD
100 : 108 : 112 : 126 : 25
100 : 94 : 96 : 90 : 31
anti-GAPDH
36KD
100 : 109 : 112 : 96 : 98
100 : 75 : 52 : 63 : 81

Figure S4C

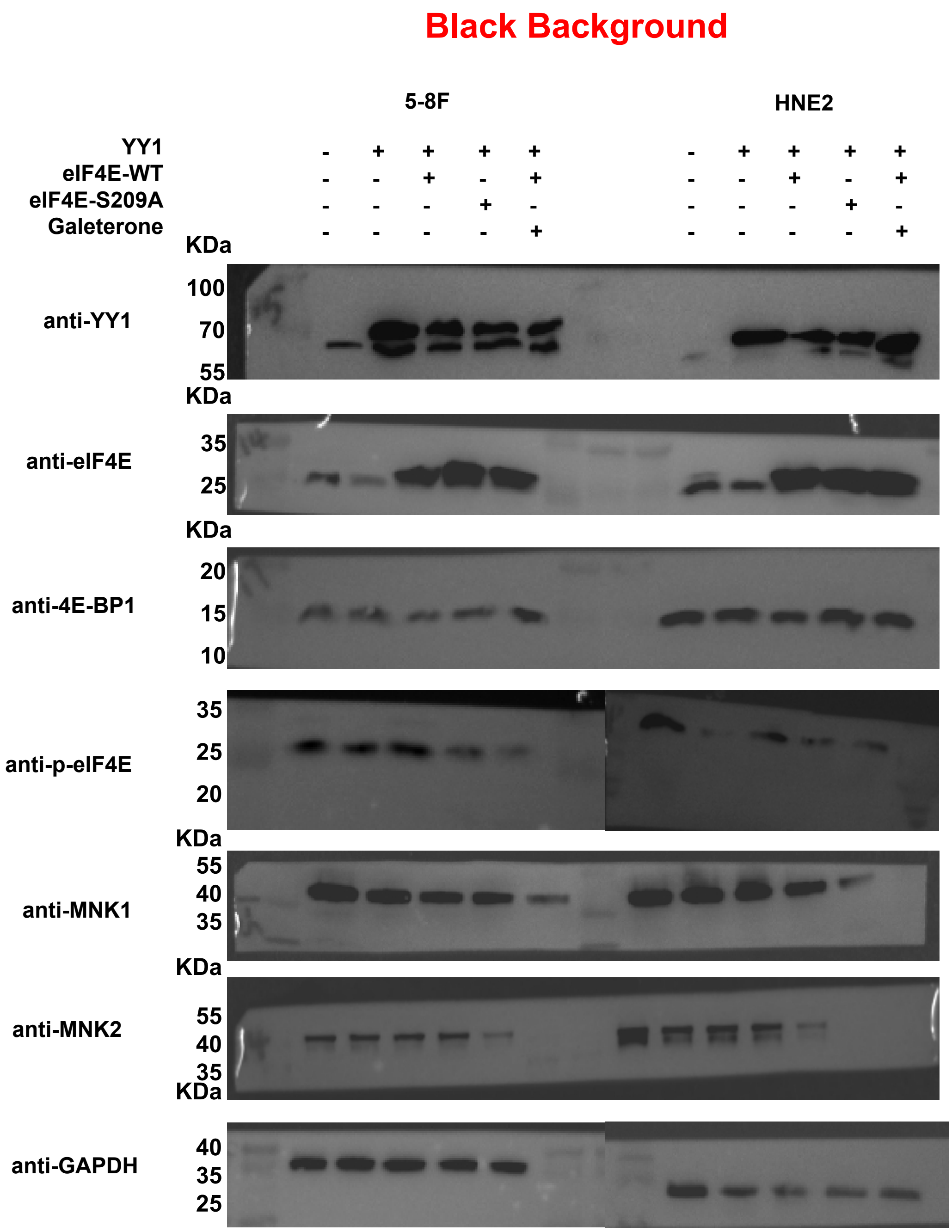
Black Background
5-8F
HNE2
YY1
eIF4E-WT
eIF4E-S209A
Galeterone
KDa
anti-YY1
100
70
55
anti-eIF4E
35
25
anti-4E-BP1
20
15
10
anti-p-eIF4E
35
25
20
anti-MNK1
55
40
35
anti-MNK2
55
40
35
anti-GAPDH
40
35
25
