# Supplementary material for: YY1 Suppresses Tumor Growth by Negatively Regulating the Expression and Translation Activity of eIF4E in Nasopharyngeal Carcinoma
Source: Cancers (Basel). 2026 Oct 9;18(20):3257. doi: 10.3390/cancers18203257 (PMC13702381; doi:10.3390/cancers18203257)
Supplement: Supplementary file 1 [file cancers-18-03257-s001.zip › cancers-4432541-Supplementary Materials.pdf]

Supplementary Materials

YY1 Suppresses Tumor Growth by Negatively Regulating the Expression and Translation Activity of eIF4E in Nasopharyngeal Carcinoma

Yumei Duan [1,2,3,4,†], Xiangting Zhou [1,2,3,†], Jianxia Wei [1,2,3], Changning Xue [1,2,3], Lemei Zheng [1,2,3], Ting Zeng [1,2,3], Hongyu Deng [1,2], Songqing Fan [5], Wei Xiong [1,2,3], Ming Zhou [1,2,3], Shuping Peng [1,2,3] and Mengna Li [1,2,3,*]

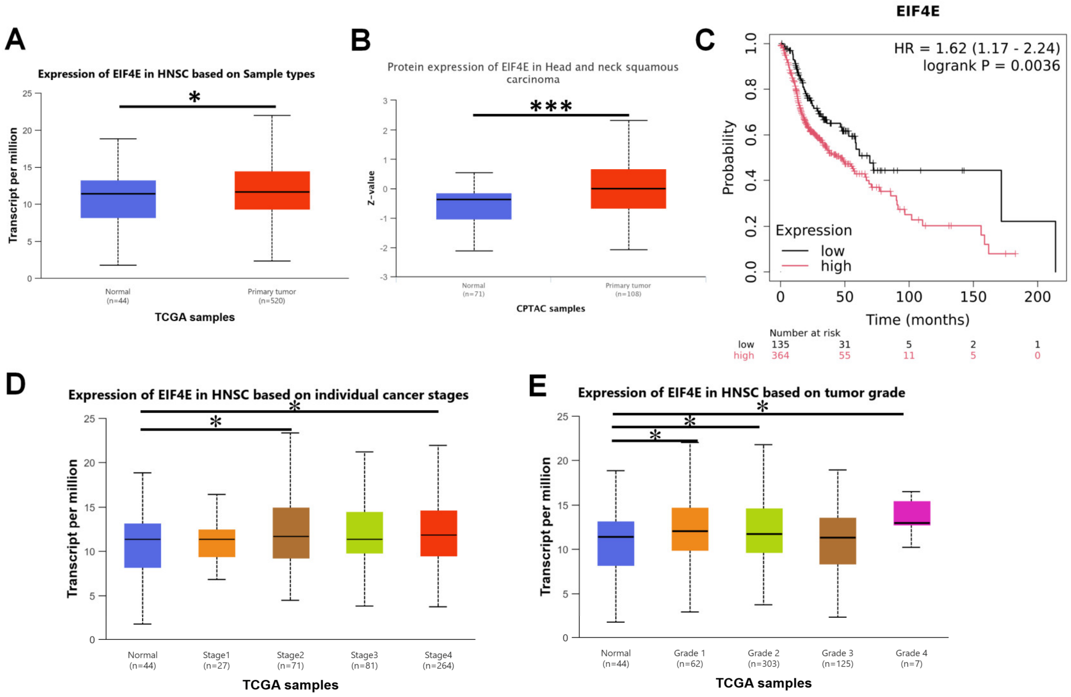

Figure S1: EIF4E is high expression in HNSC. A The mRNA expression of eIF4E in HNSC by UALCAN database. B eIF4E protein expression in HNSC by CPTAC database. C The overall survival curve of eIF4E in HNSC by the Kaplan–Meier plotter database. D Analysis of the individual cancer stages of eIF4E in HNSC by UALCAN database. E Analysis of the tumor grade of eIF4E in HNSC by UALCAN database.

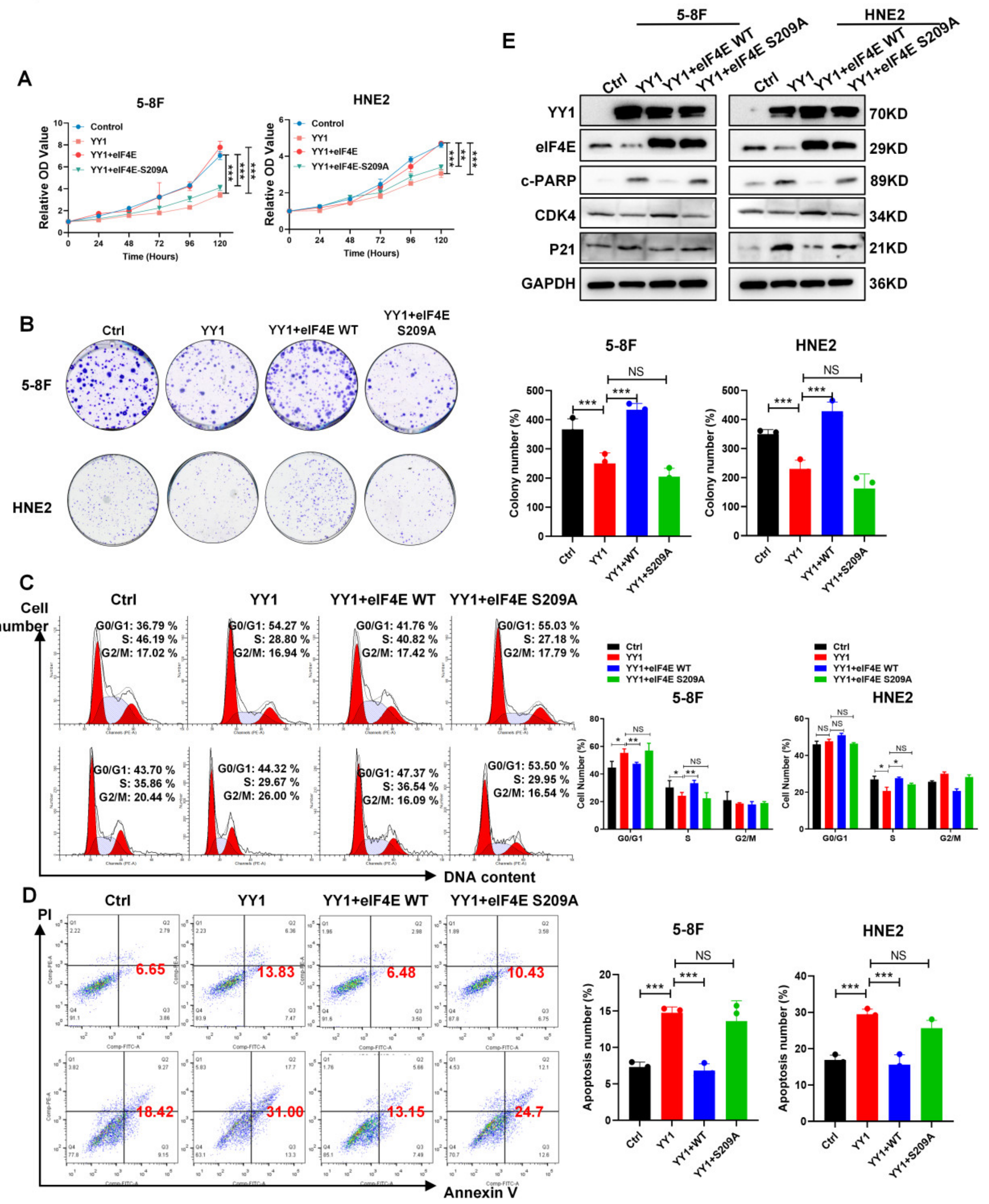

Figure S2: The promoting effect on the proliferation of NPC cells depends on the activation of eIF4E S209. A CCK-8 analysis of cell proliferation in 5-8F and HNE2 cell lines stably with YY1 overexpression, YY1 and eIF4E(WT) simultaneous overexpression, YY1 and eIF4E(S209A) simultaneous overexpression or control, respectively. B Cloning formation analysis of these four cell lines(left), percents of the colony number of the four groups (right). C Cell cycle of cells were determined by FACS compared with the control group(left), quantification of cells in different stages of these four cell lines (right). D Cells were dyed with Annexin V-FITC and PI to track the apoptosis (left), quantification of the apoptosis cells of these four cell lines (right). E Western blot showed significantly expressed proteins involved in cell proliferation (CDK4 and P21) and cell apoptosis (c-PARP) in these four cell lines. GAPDH served as an internal control. Error bars represented the mean ± SD. ***P* <0.01; ****P* <0.001; NS, no significance.

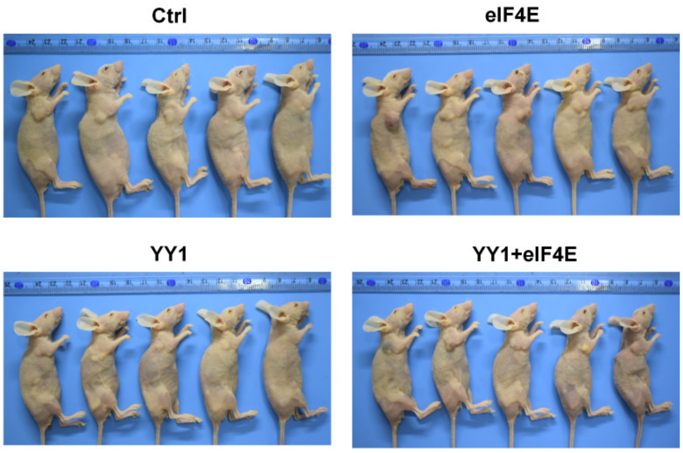

Figure S3: YY1 inhibits tumor growth by down-regulating eIF4E expression in vivo. Representative images of the 5-8F xenograft model in nude mice (n= 5).

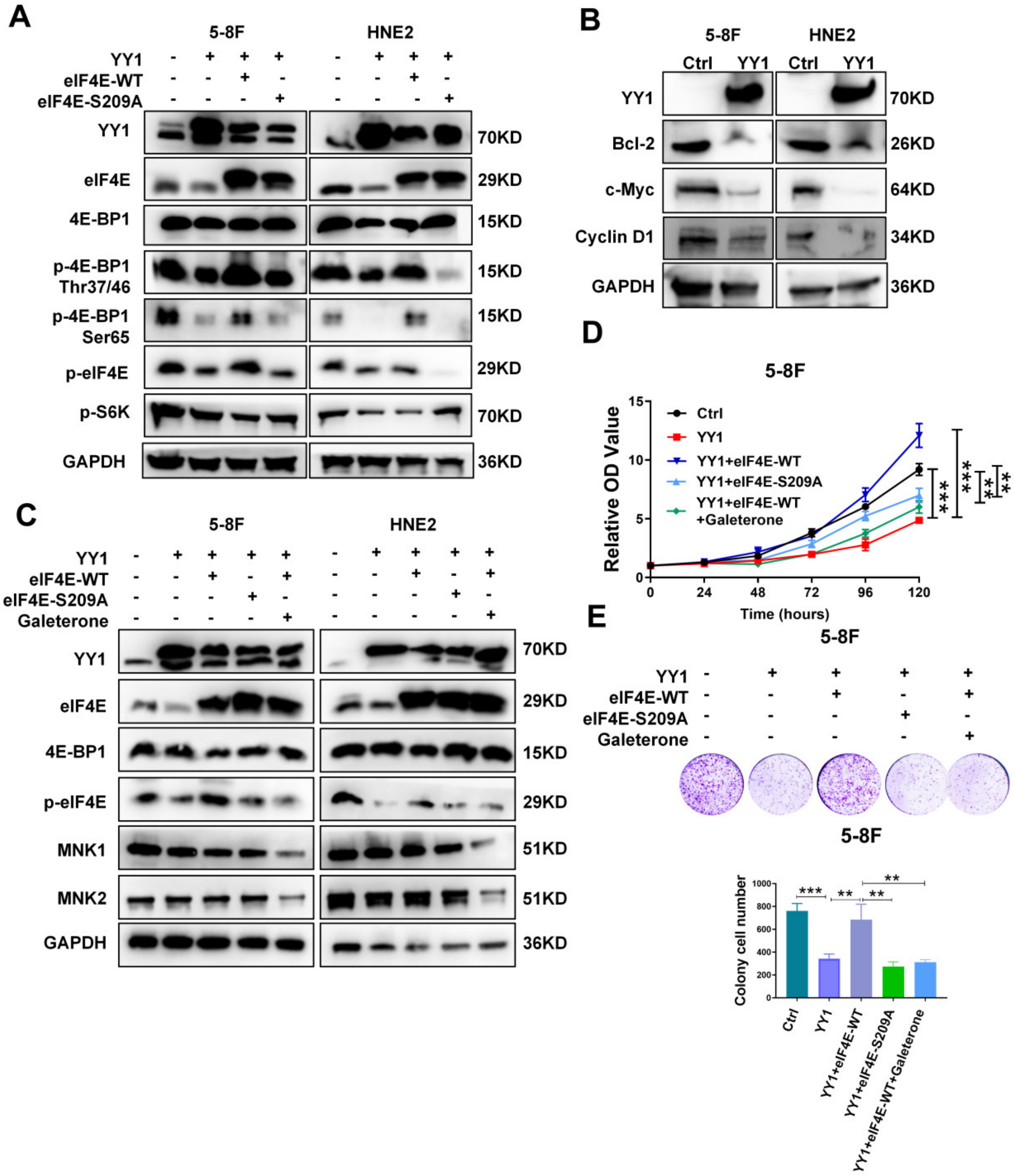

Figure S4: YY1 suppresses NPC cell growth by inhibiting eIF4E phosphorylation and translation activity. A Western blot analysis was performed to detect YY1, phosphorylated eIF4E, total eIF4E, 4E-BP1 phosphorylated at Thr37/46 and Ser65, total 4E-BP1, and phosphorylated S6K. GAPDH was used as an internal control. B Western blot analysis was used to detect the expression of YY1, Bcl-2, c-Myc, and cyclin D1. GAPDH served as an internal control. C Western blot analysis was performed to examine MNK1, MNK2, YY1, p-eIF4E, total eIF4E, and 4E-BP1. GAPDH was used as internal control. D CCK-8 assay was performed to detect the cell viability of 5-8F cells treated with Galeterone. E Colony-forming assay was used to detect the cell growth of 5-8F cells treated with Galeterone. Error bars represented the mean ± SD. **P <0.01; ***P <0.001.
